# Transcriptome data on maternal RNA of 24 individual zebrafish eggs from five sibling mothers^☆^

**DOI:** 10.1016/j.dib.2016.04.045

**Published:** 2016-04-26

**Authors:** Johanna F.B. Pagano, Han Rauwerda, Wim C. de Leeuw, Paul Wackers, Mark de Jong, Wim Ensink, Rob Dekker, Ulrike Nehrdich, Herman P. Spaink, Martijs Jonker, Timo M. Breit

**Affiliations:** a RNA Biology & Applied Bioinformatics research group, Swammerdam Institute for Life Sciences, Faculty of Science, University of Amsterdam, Amsterdam, The Netherlands; b Institute of Biology Leiden, Faculty of Science, Leiden University, Leiden, The Netherlands

**Keywords:** Zebrafish, Danio rerio, Egg transcriptome, Single egg

## Abstract

Maternal mRNA that is present in the mature oocyte plays an important role in the proper development of the early embryo. To elucidate the role of the maternal transcriptome we recently reported a microarray study on individual zebrafish eggs from five different clutches from sibling mothers and showed differences in maternal RNA abundance between and within clutches, “**Mother-specific signature in the maternal transcriptome composition of mature, unfertilized Eggs**” [1]. Here we provide in detail the applied preprocessing method as well as the R-code to identify expressed and non-expressed genes in the associated transcriptome dataset. Additionally, we provide a website that allows a researcher to search for the expression of their gene of interest in this experiment.

**Specifications Table**

**Table:** 

Subject area	*Biology*
More specific subject area	*Developmental biology*
Type of data	*Figures, R-code*
How data was acquired	DNA microarray scanner G2565CA (Agilent Technologies) using Agilent Feature Extraction software version 10.7.3.1
Data format	*Raw, normalized*
Experimental factors	Mature non treated eggs from different mothers
Experimental features	Expression profiles from 24 individual zebrafish eggs from five different clutches (mothers that are siblings).
Data source location	*University of Amsterdam, The Netherlands*
Data accessibility	Data are within this article and *via accession number GEO: GSE72839 at*http://www.ncbi.nlm.nih.gov/geo/query/acc.cgi?acc=GSE72839*, and*http://rnabiology.nl/Dr-Browser.html

**Value of the data**

• This data contains the maternal transcriptomes of 24 individual zebrafish eggs, whereas up to date only a very limited amount of data is available on transcriptomes of individual eggs.
• In this dataset, maternal transcriptomes from several individual eggs from the same mother are available; an experimental design that makes this dataset unique.
• This data offers a valuable and searchable resource on maternal gene expression and can be used for zebrafish embryology studies.

## 1 Data

The data that are shared here include *the raw data of the experiment, via Geo submission GEO: GSE72839 (raw data: Agilent data extraction files, as well as the normalized data together with the experiment design).*

*Data on the definition of expressed and non-expressed genes, together with the relevant R-code are also provided, as well as the plots of expression levels of expressed genes via a searchable web interface, which also allows to detect the set of genes that have a similar expression profile as the gene of interest.*

*See also* http://www.ncbi.nlm.nih.gov/geo/query/acc.cgi?acc=GSE72839 and http://rnabiology.nl/Dr-Browser.html

## 2 Experimental design, materials and methods

The basic material and methods, including “zebrafish eggs”, “RNA extraction & microarrays” are presented in the manuscript describing the original finding of the zebrafish oocyte study [1].

### 2.1 Data preprocessing and normalization

In order to distinguish expressed transcripts from non-expressed transcripts the procedure described in Supplemental File SD1 has been applied to the raw data (the separate microarray data is combined in Supplemental File SD2 and Supplemental File SD3; the annotation file is in Supplemental File SD4). In short, for each microarray probe the log variance of the log intensity over the entire experiment of the Cy3 channel was determined. In the resulting bimodal distribution (Supplemental Fig. SF1) the log variance between the low and the high-variance peak was determined at −3.74 with the lowest number of microarray probes below which probes were labeled ‘low-variance probes’ (*n*=58,212) and above which probes were labeled ‘high-variance probes’ (*n*=30,512). As expected, probes in the low-variance distributions have in general lower intensities than probes in the high-variance distribution (Supplemental Fig. SF2). Hence, the probability of a probe to be part of the high- or the low-variance distribution depends on the intensity level of that probe. Now we can infer per array, per probe the conditional probability Pr(E|I) of being in the high-variance distribution given a certain intensity. This conditional probability is calculated from the intensity probabilities of the two distributions by applying Bayes׳ theorem:

(1) $$\mathrm{Pr}(E|I)=\frac{\mathrm{Pr}\;(I|E)∙\mathrm{Pr}\;(E)}{\mathrm{Pr}(I|E)∙\mathrm{Pr}\left(E\right)+\mathrm{Pr}\;(I\mathrm{|\neg}E)∙\mathrm{Pr}\;(\neg E)}$$

With Pr(E) as the probability of a probe being in the high-variance distribution, Pr(I) as the probability of a probe having a certain intensity value

Probabilities are calculated by dividing the intensity range into bins of 0.25 log2-intensity units. In order to avoid erratic behavior in lowly populated bins the lower intensity region has been collapsed into one bin. Because the conditional probability Pr(E^|^I) will increase with increasing intensities we can determine a per array intensity level above which we have a certain confidence that probes will belong to the high-variance distribution. In other words: an intensity level above which we believe that signals do not originate from noise. It is noteworthy that, because the distributions are overlapping, also low-variance probes with high intensities are included. Here the per-microarray threshold to label a probe (or transcript) as “expressed” was set at a likelihood of larger than 0.95. With this threshold, interpreted as the intensity above which we believe a probe is reliably measured on a specific array, we are stringently avoiding false positives. Both per array intensity cut-off value, as the per array likelihood of being expressed as a function of intensity can be found in Supplemental Fig. SF3. To call a transcript “expressed somewhere in the experiment” we applied a second threshold, i.e. the requirement that a specific probe must be assigned “expressed” in at least four samples. Finally, Ensembl transcripts identifiers were linked to their Ensembl gene identifiers where a gene was labeled “expressed” when at least one of its containing transcripts was called “expressed somewhere in the experiment”. The resulting two data sets with expressed and non-expressed probes are in Supplemental File SD5 and Supplemental File SD6, respectively.

The quality of the microarray data was assessed via multiple quality-control checks, i.e. visual inspection of the scans, testing against criteria for foreground and background signals, testing for consistent performance of the labeling dyes, checking for spatial effects through pseudo-color plots, and inspection of pre- and post-normalized data with box plots, ratio-intensity (RI) plots and PCA plots. All microarrays passed the minimal criteria for quality assessment of the microarray data and were used in the analyses. Handling, analysis and visualization of all data was performed in R (http://cran.r-project.org/) using the Bioconductor packages affy, limma and maanova [2]. In this stage, we removed two genes (rdh14b and zgc:63480) from the dataset, due to the fact that they each had an extreme high expression value in one of the samples, whereas the expression was consistently absent in all other samples, which resulted in an unrealistic fold change of over 3,000 times. Log2 transformed Cy3 data was normalized between arrays by quantile normalization from the robust multi-array average (RMA) function in the R Bioconductor affy package, resulting in the normalized data as deposited in GEO data set GEO:GSE72839.

### 2.2 Search and visualization of expressed genes

Via the web site http://rnabiology.nl/Dr-Browser.html the set of expressed genes, as given in Supplemental File SD5 can be browsed and searched. An in-text search is possible on Ensembl identifiers and linked Refseq, Entrez, ZFIN and Unigene identifiers, as well as on the descriptions and symbols of the genes. Also, for each gene of interest, the associated set of 100 genes with the most similar gene expression, based on correlation can be selected.
