# Supplementary material for: Transcriptome data on maternal RNA of 24 individual zebrafish eggs from five sibling mothers
Source: Data Brief. 2016 Apr 26;8:69–72. doi: 10.1016/j.dib.2016.04.045 (PMC4887590; doi:10.1016/j.dib.2016.04.045)
Supplement: Supplementary file 2 — Supplementary material [file mmc2.pdf]

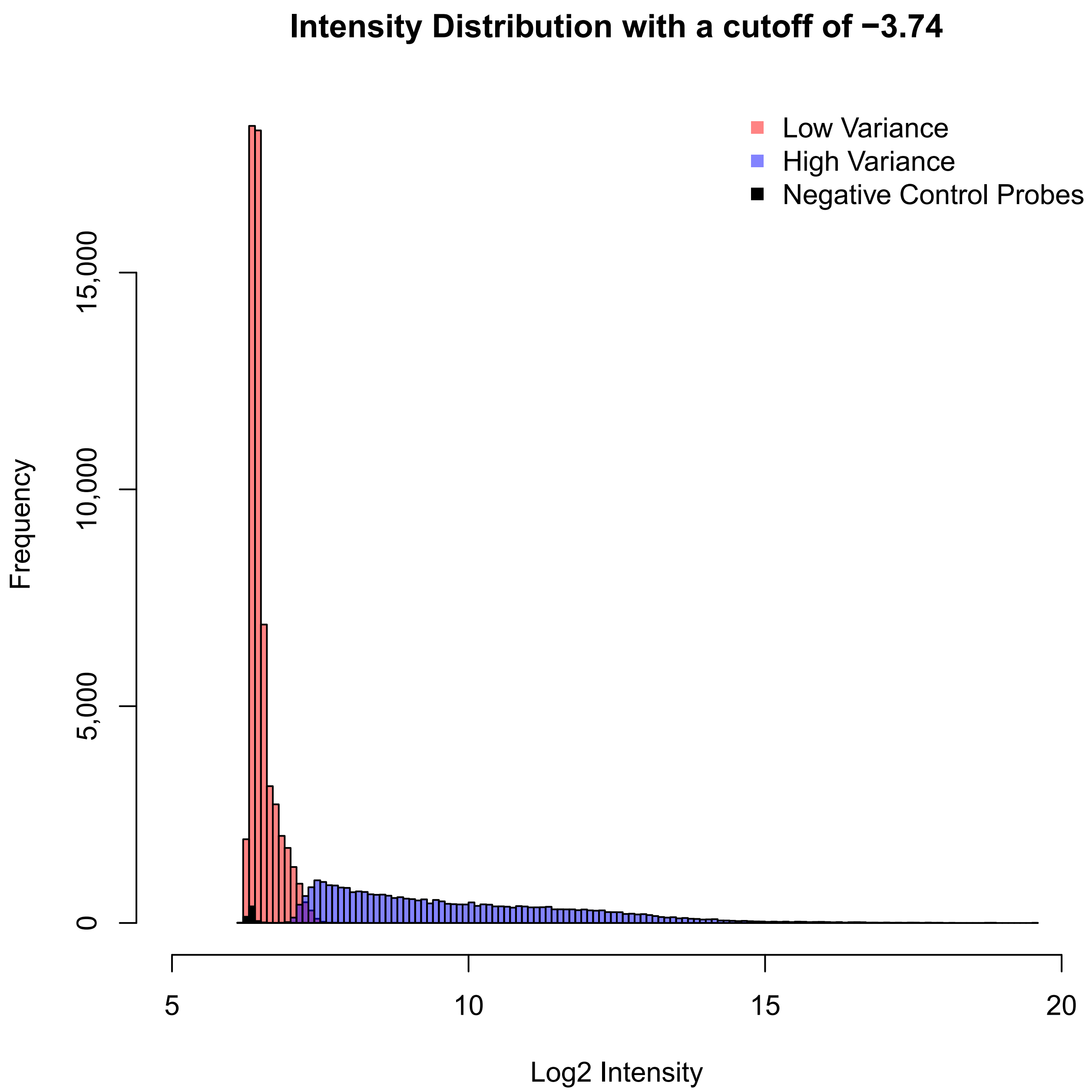

Supplemental Figure SF2
Distribution of the log2 intensity values of probes with a log variance repectively below (low variance probes) and above (high variance probes) the cutoff of -3.74, and of the negative control probes.
