# Supplementary material for: Transcriptome data on maternal RNA of 24 individual zebrafish eggs from five sibling mothers
Source: Data Brief. 2016 Apr 26;8:69–72. doi: 10.1016/j.dib.2016.04.045 (PMC4887590; doi:10.1016/j.dib.2016.04.045)
Supplement: Supplementary file 3 — Supplementary material [file mmc3.pdf]

Supplemental Figure SF3 (2 x 24 pages)
Page A. Conditional probabilities (Pr(E|I)) per intensity bin of 0.25 log2 intensity units calculated for the indicated egg.
Page B. Histogram of log2 intensity values of low-variance and high-variance probes for the indicated egg. The height of the bars indicate the number of probes present in a bin of 0.25 log2 intensity units. The cut-off above which a probe is considered to be expresses in this egg is indicated by a vertical black line.

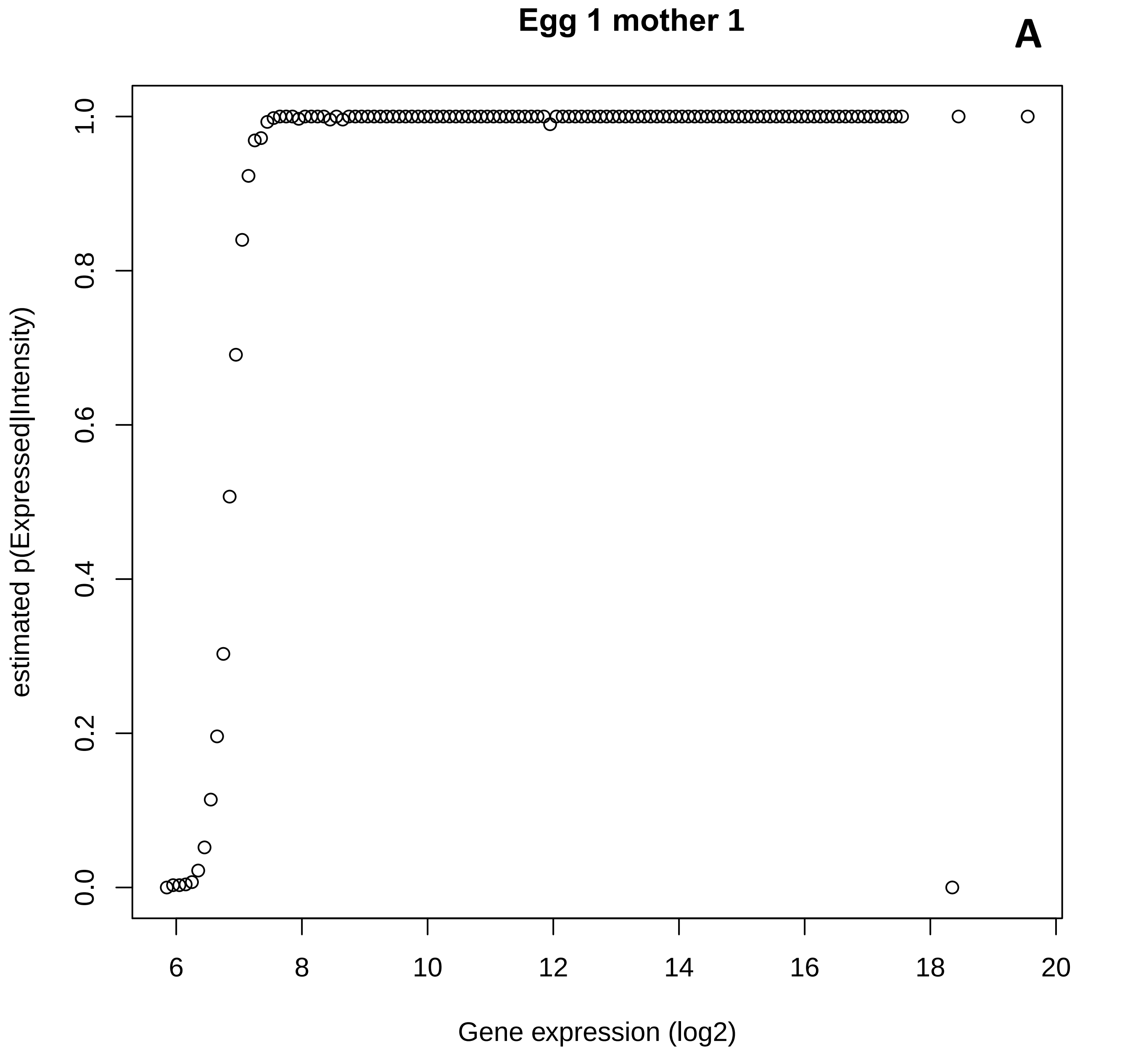
Egg 1 mother 1
A
estimated p(Expressed|Intensity)
0.0
0.2
0.4
0.6
0.8
1.0
6
8
10
12
14
16
18
20
Gene expression (log2)

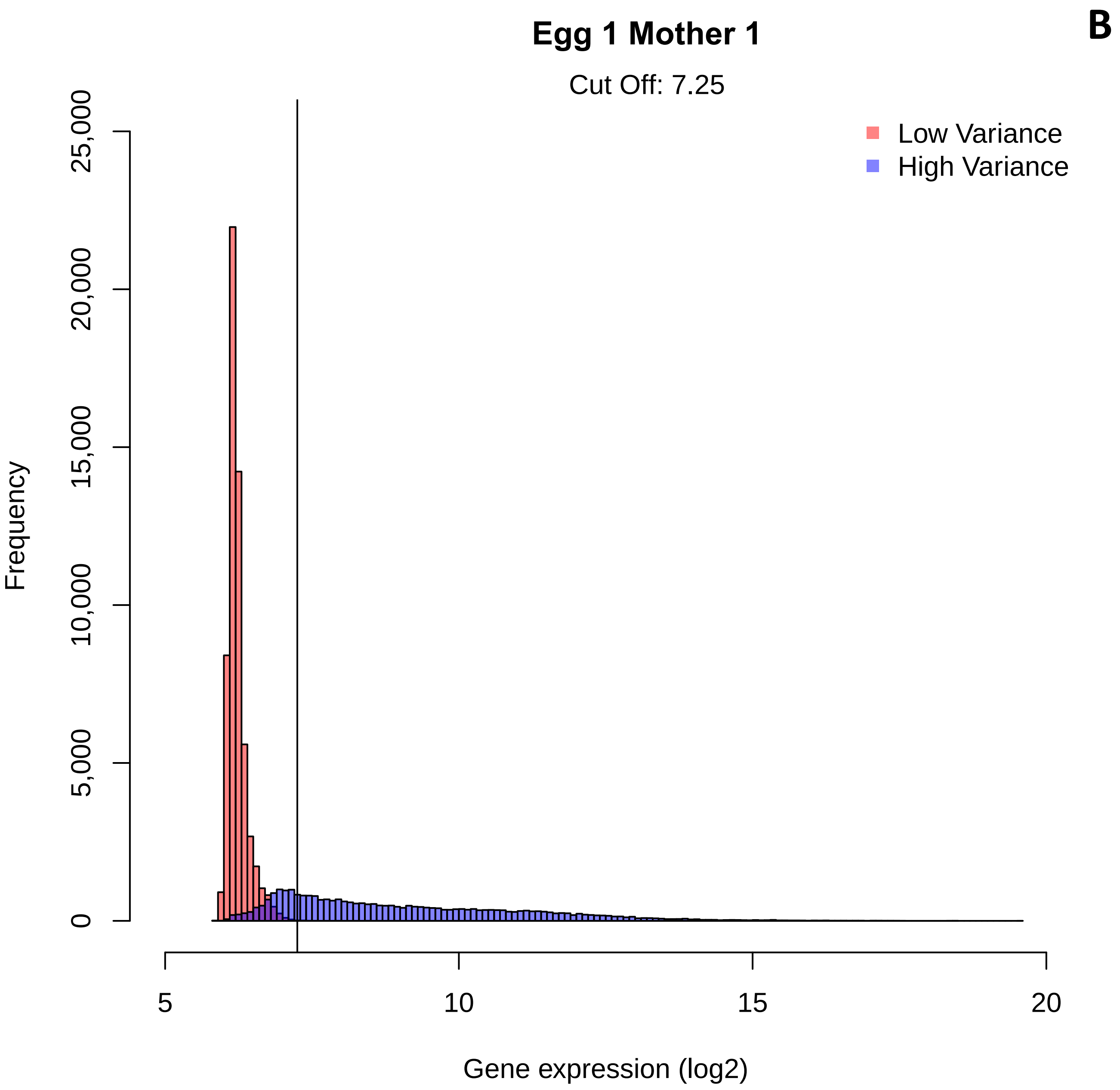
B
Egg 1 Mother 1
Cut Off: 7.25
Low Variance
High Variance
Frequency
0
5,000
10,000
15,000
20,000
25,000
5
10
15
20
Gene expression (log2)

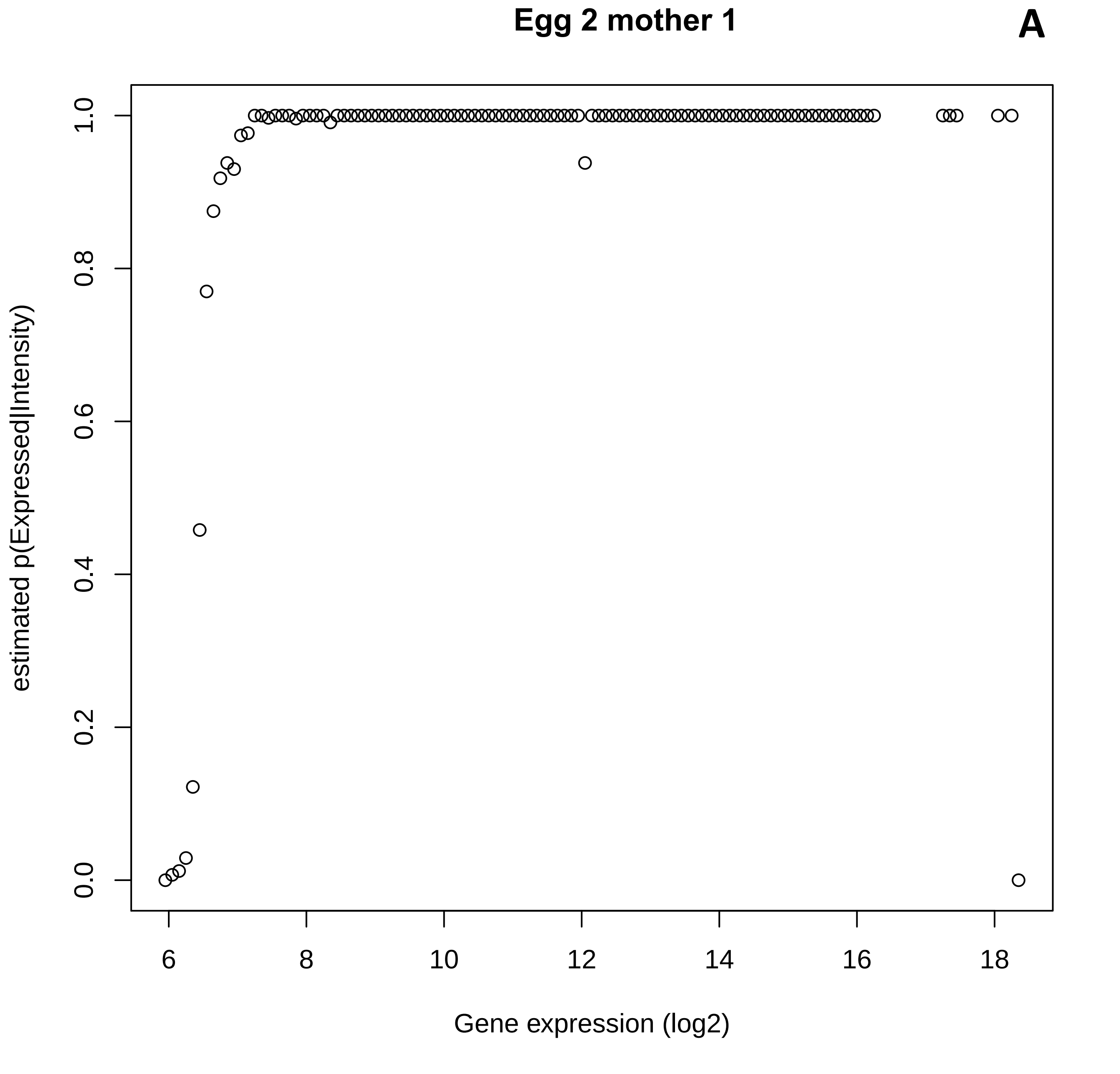
Egg 2 mother 1
A
estimated p(Expressed|Intensity)
0.0
0.2
0.4
0.6
0.8
1.0
6
8
10
12
14
16
18
Gene expression (log2)

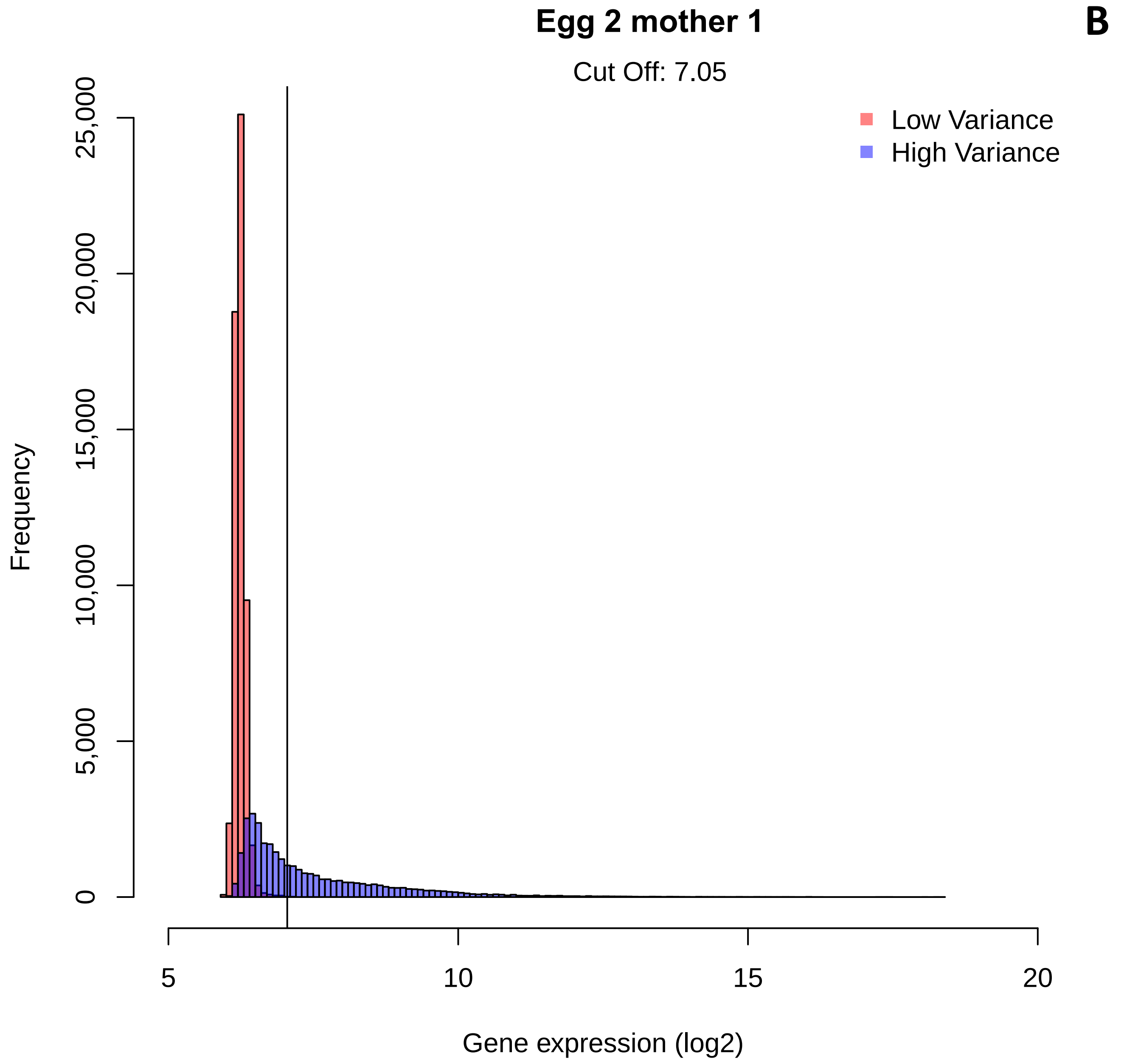

Egg 2 mother 1
B
Cut Off: 7.05
Low Variance
High Variance
Frequency
0
5,000
10,000
15,000
20,000
25,000
5
10
15
20
Gene expression (log2)

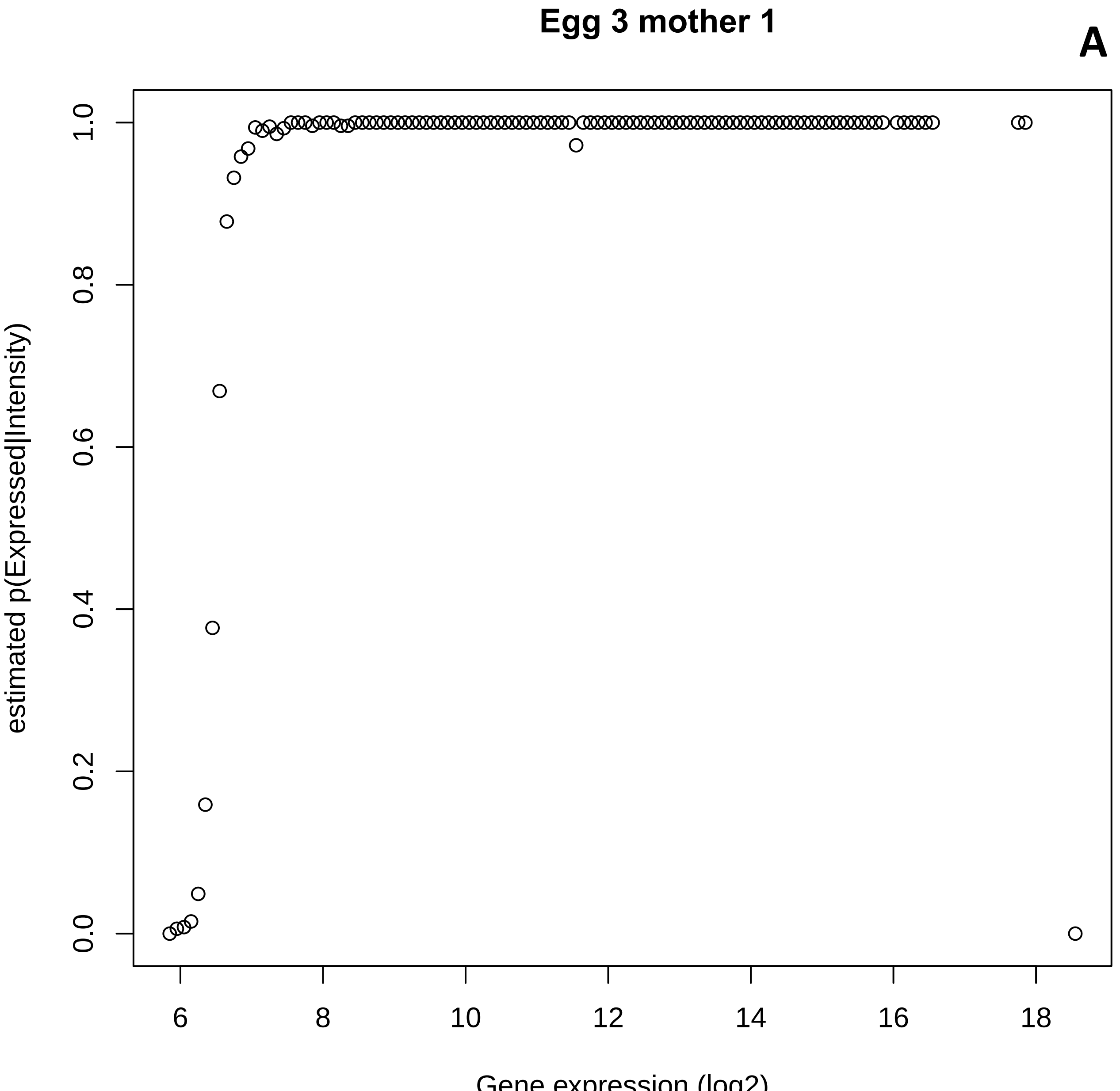

Egg 3 mother 1
A
estimated p(Expressed|Intensity)
0.0
0.2
0.4
0.6
0.8
1.0
6
8
10
12
14
16
18
Gene expression (log2)

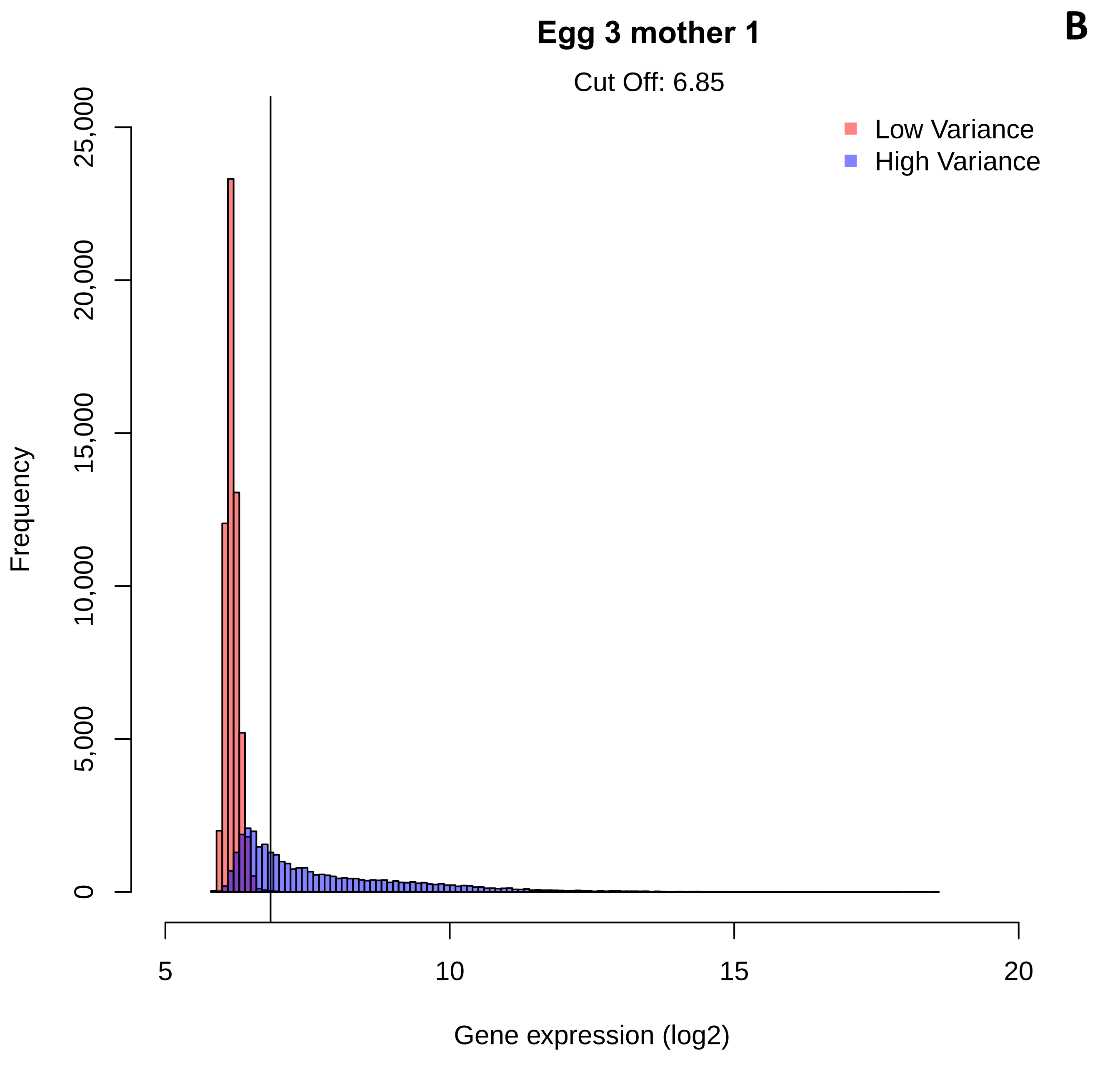
B
Egg 3 mother 1
Cut Off: 6.85
Low Variance
High Variance
Frequency
0
5,000
10,000
15,000
20,000
25,000
5
10
15
20
Gene expression (log2)

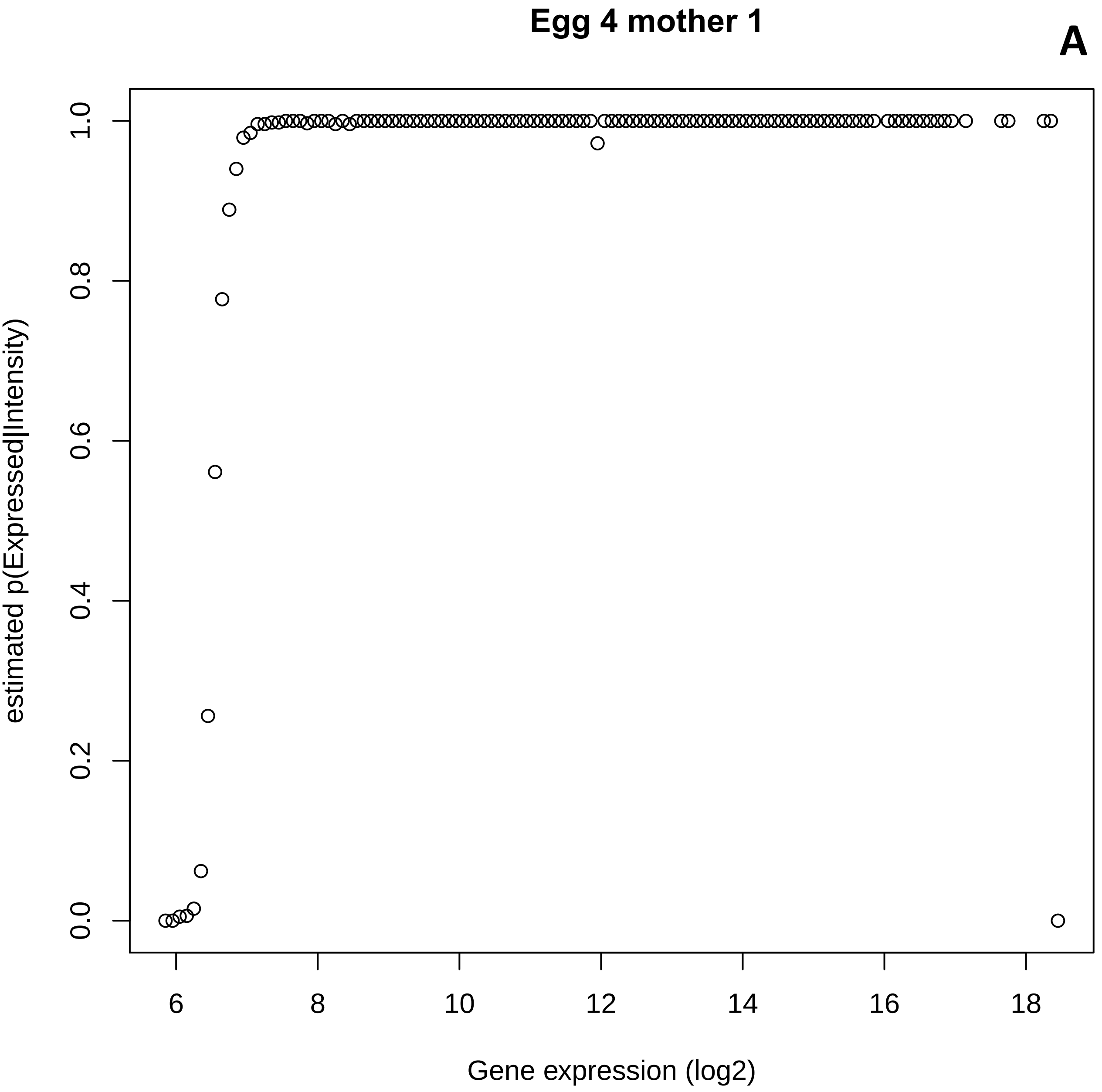

Egg 4 mother 1
A
estimated p(Expressed|Intensity)
0.0
0.2
0.4
0.6
0.8
1.0
6
8
10
12
14
16
18
Gene expression (log2)

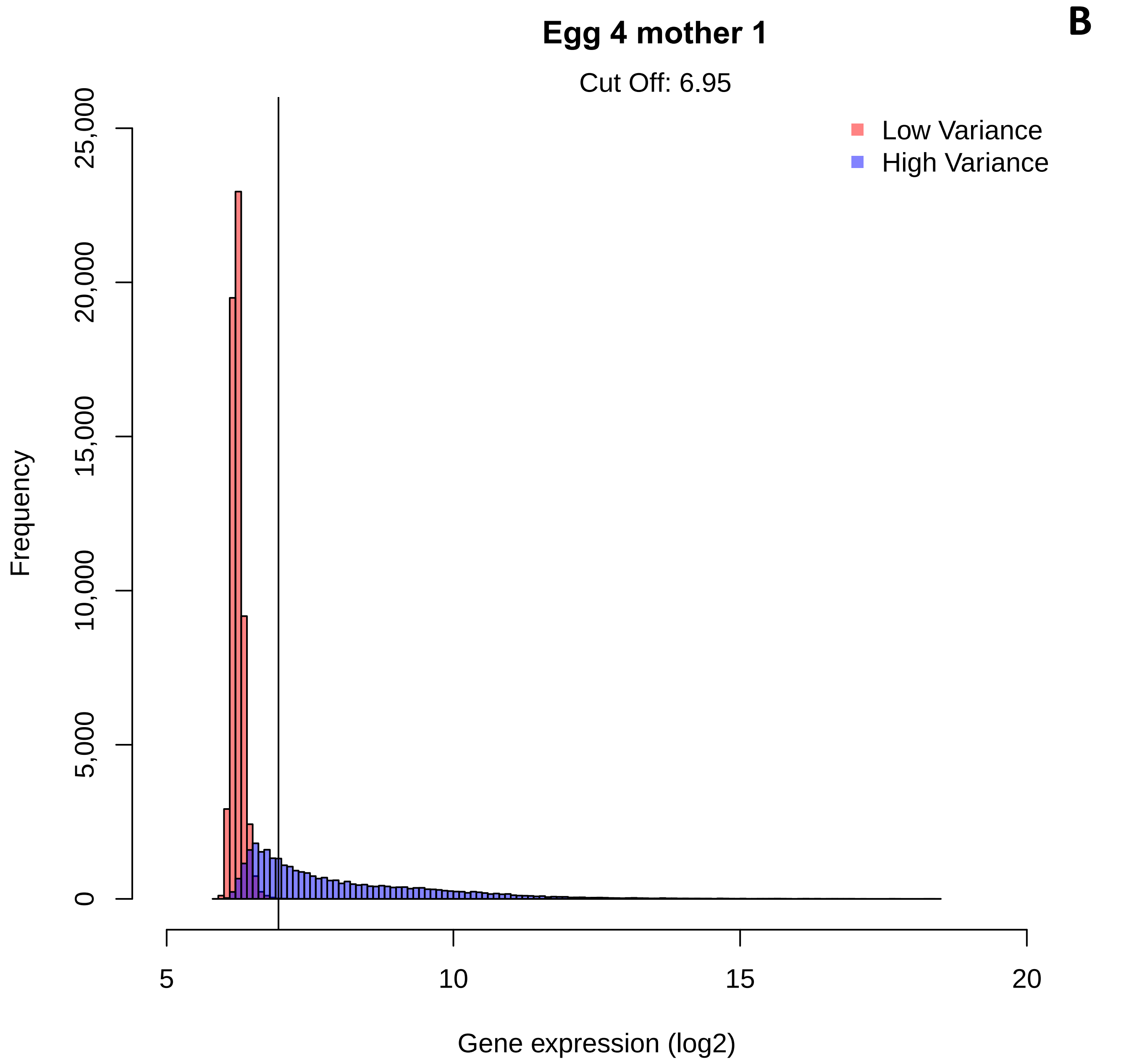
B
Egg 4 mother 1
Cut Off: 6.95
Low Variance
High Variance
Frequency
0
5,000
10,000
15,000
20,000
25,000
5
10
15
20
Gene expression (log2)

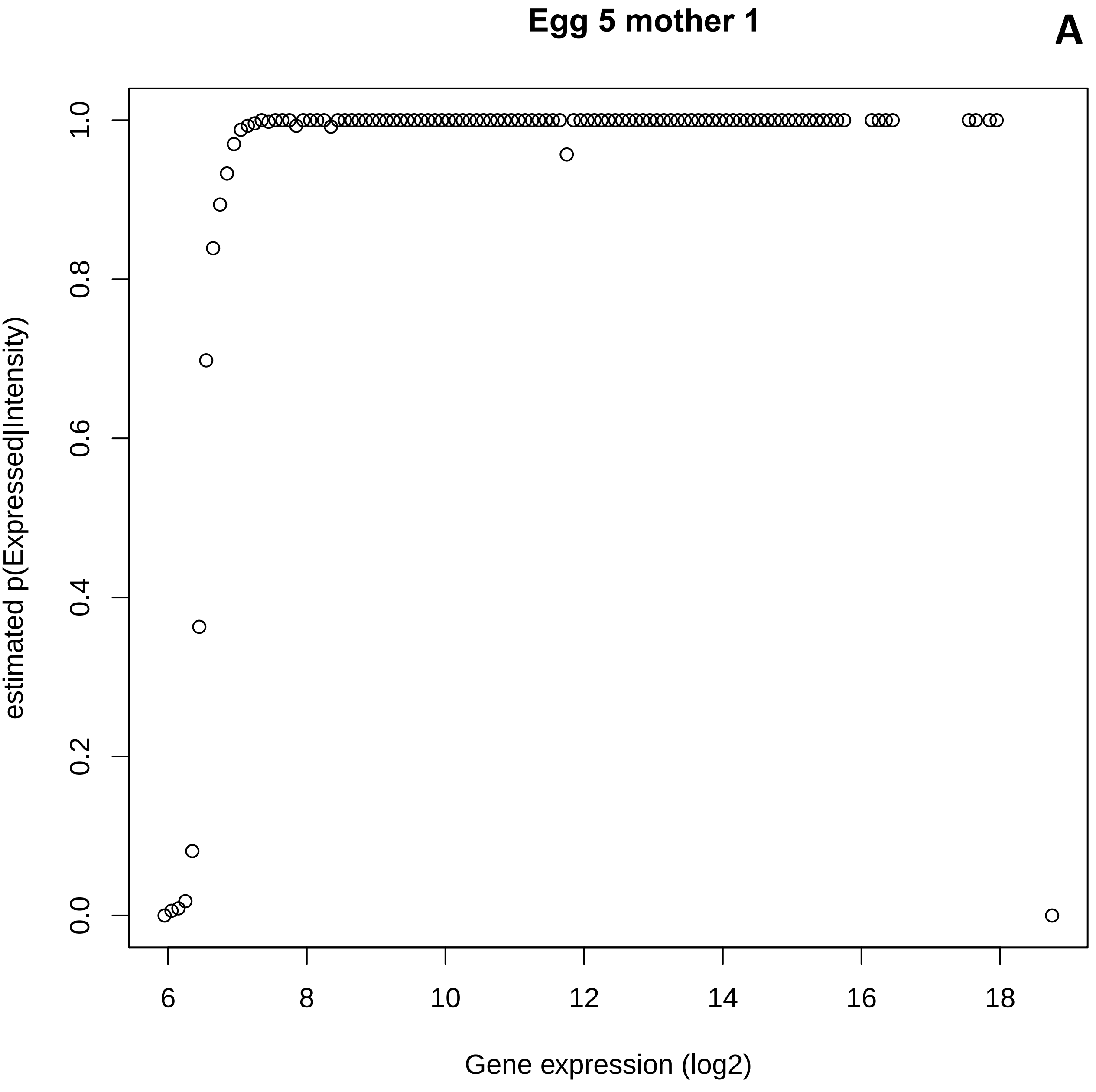

Egg 5 mother 1
A
estimated p(Expressed|Intensity)
0.0
0.2
0.4
0.6
0.8
1.0
6
8
10
12
14
16
18
Gene expression (log2)

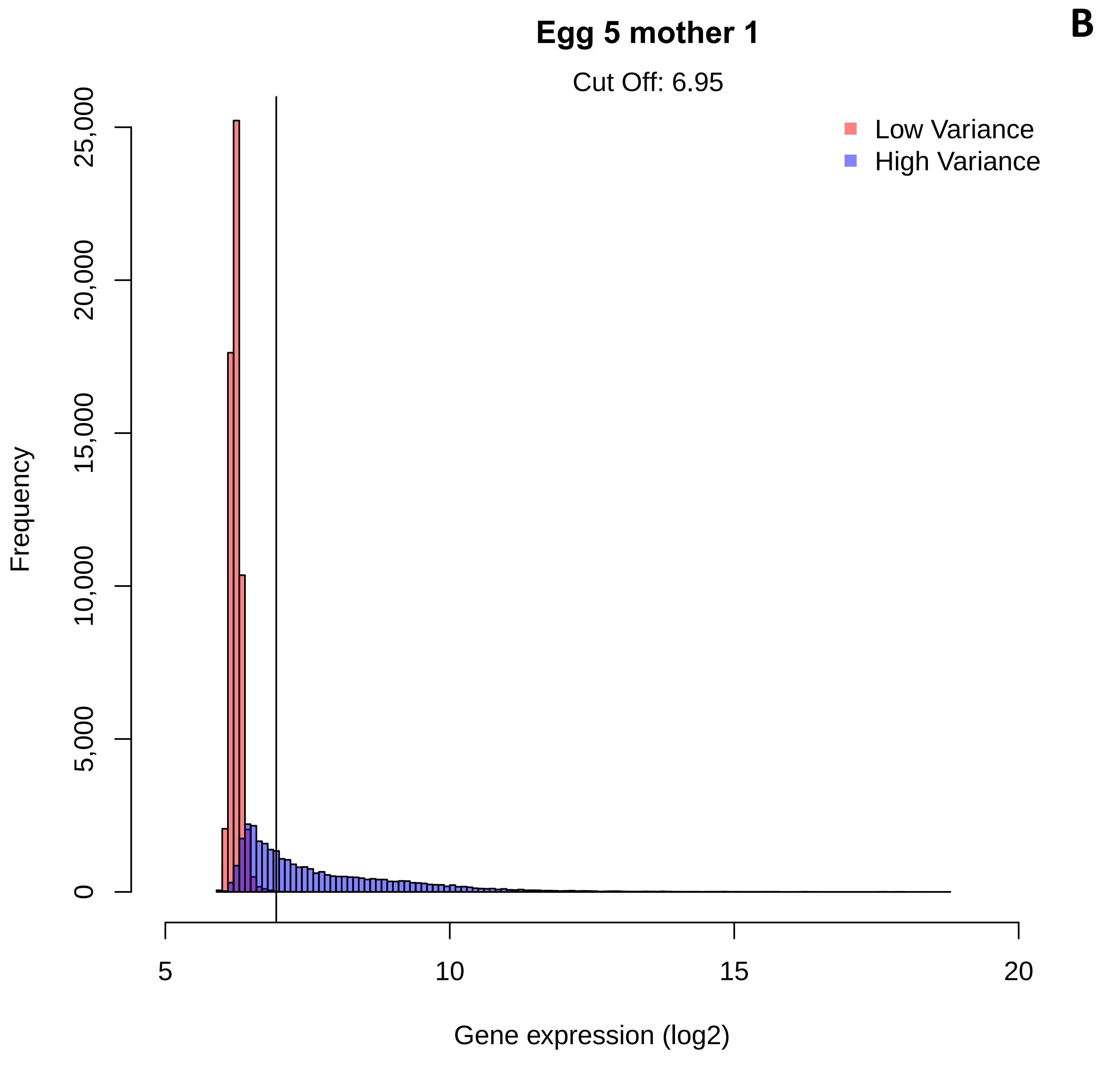

B
Egg 5 mother 1
Cut Off: 6.95
Low Variance
High Variance
Frequency
0
5,000
10,000
15,000
20,000
25,000
5
10
15
20
Gene expression (log2)

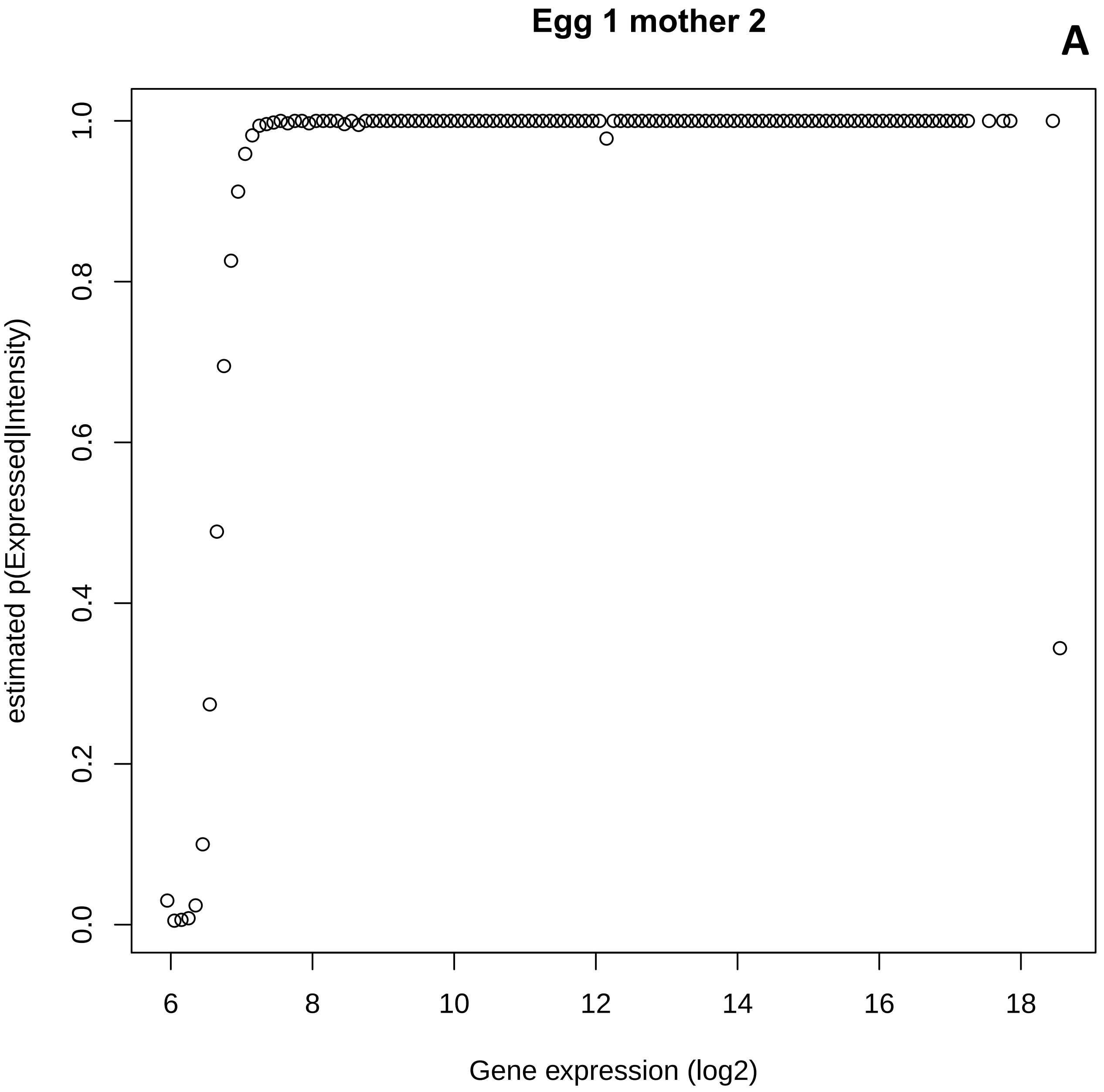

Egg 1 mother 2
A
estimated p(Expressed|Intensity)
0.0
0.2
0.4
0.6
0.8
1.0
6
8
10
12
14
16
18
Gene expression (log2)

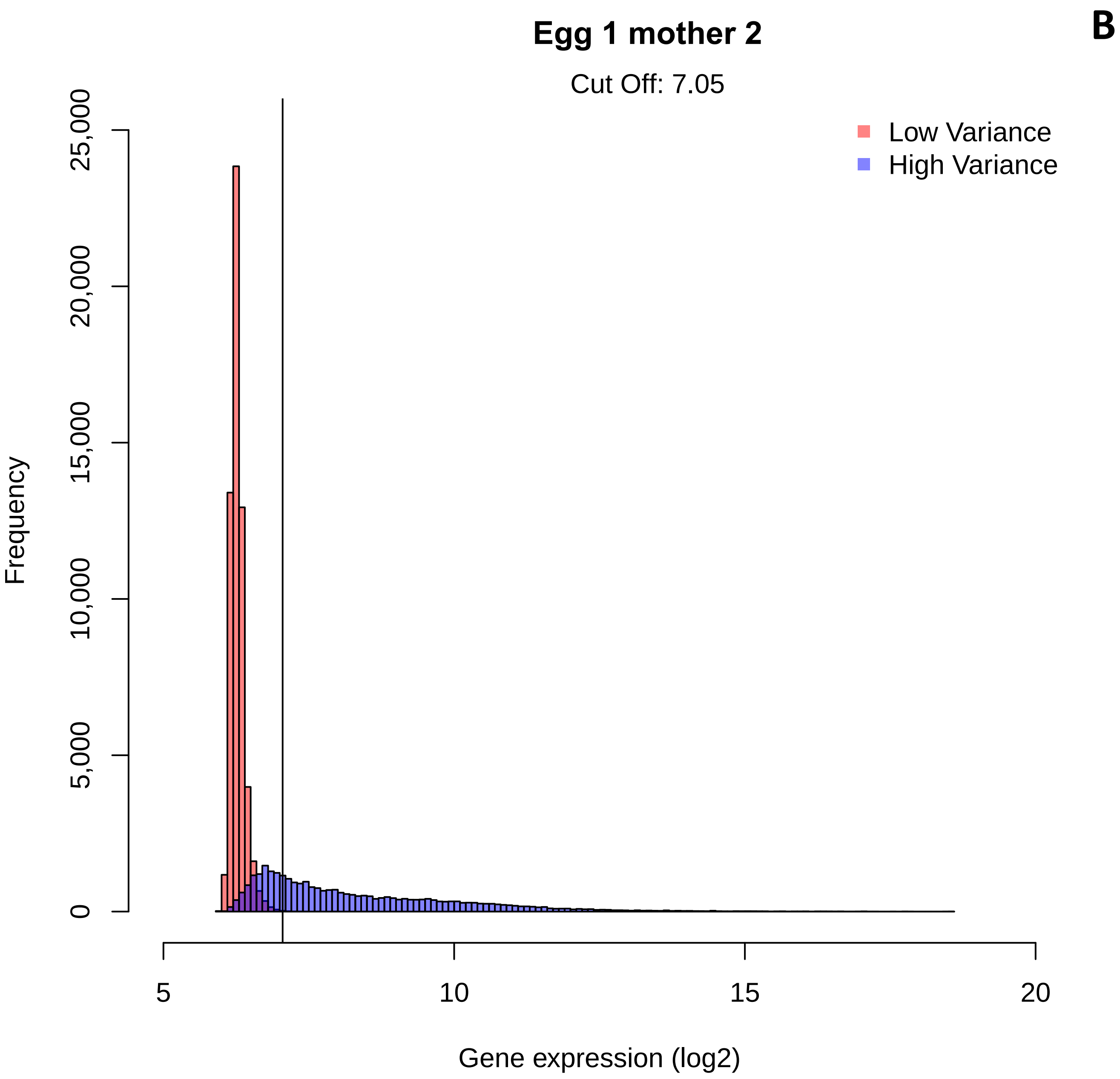
B
Egg 1 mother 2
Cut Off: 7.05
Low Variance
High Variance
Frequency
0
5,000
10,000
15,000
20,000
25,000
5
10
15
20
Gene expression (log2)

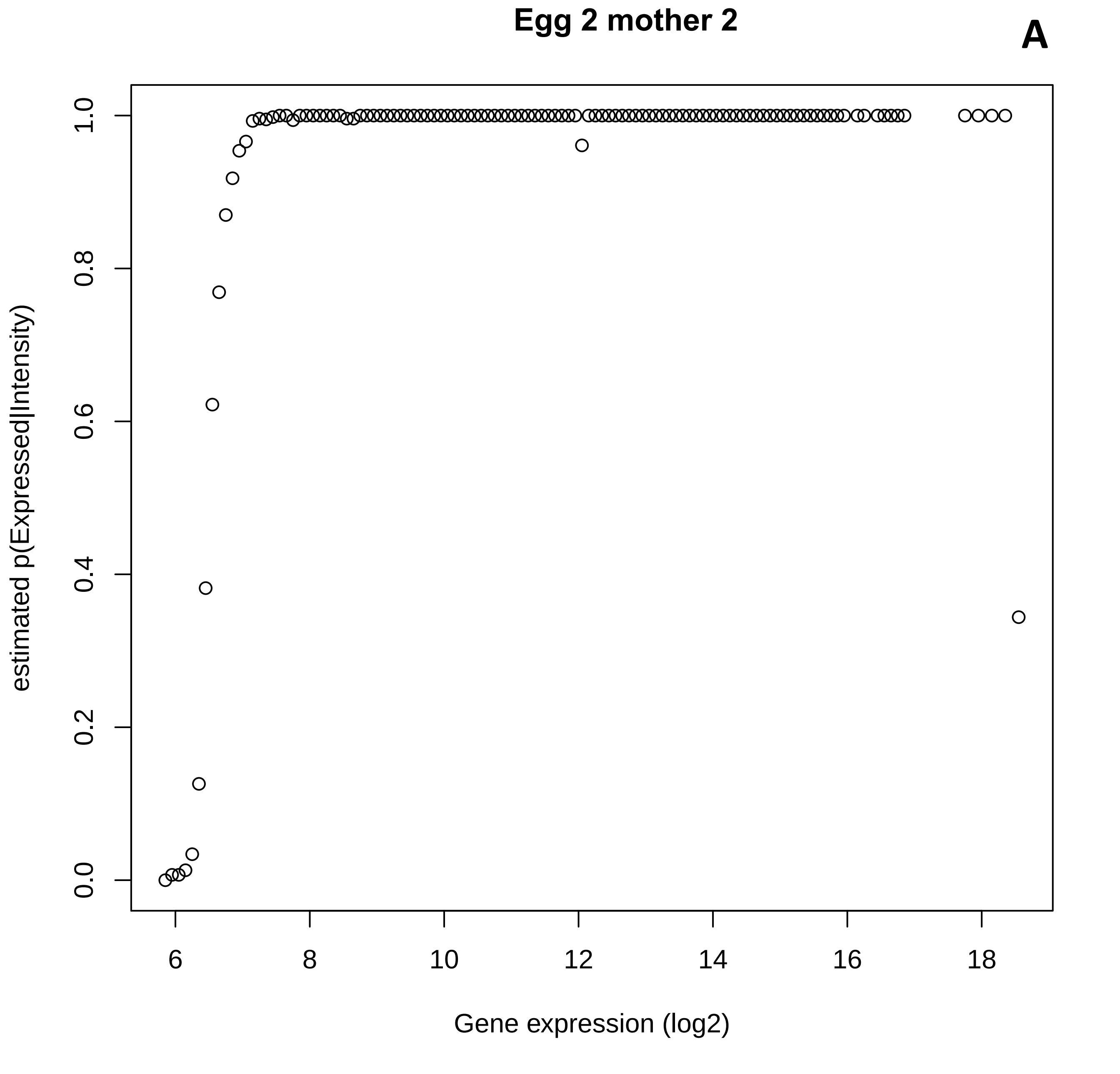

Egg 2 mother 2
A
estimated p(Expressed|Intensity)
0.0
0.2
0.4
0.6
0.8
1.0
6
8
10
12
14
16
18
Gene expression (log2)

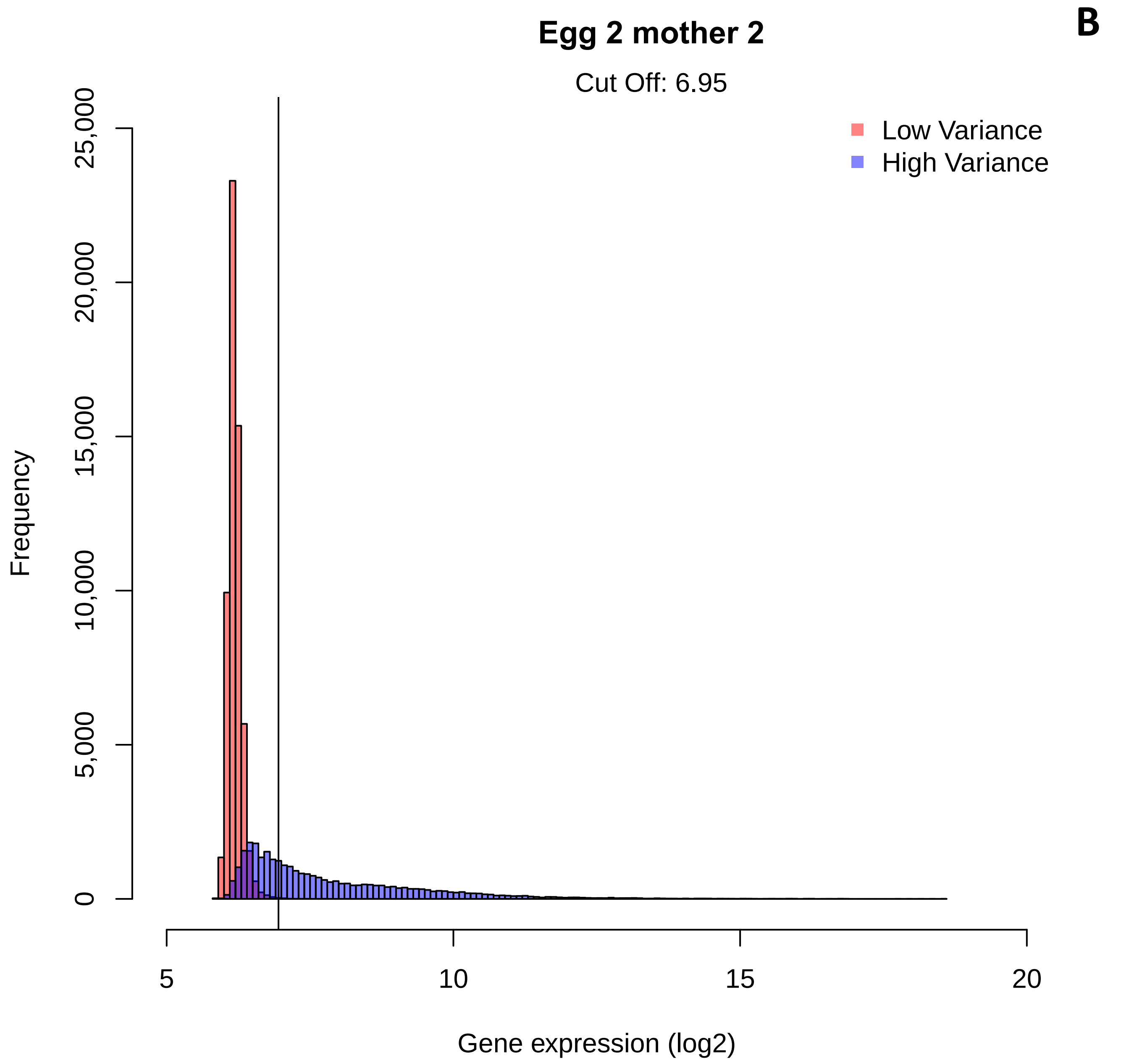
B
Egg 2 mother 2
Cut Off: 6.95
Low Variance
High Variance
Frequency
25,000
20,000
15,000
10,000
5,000
0
5
10
15
20
Gene expression (log2)

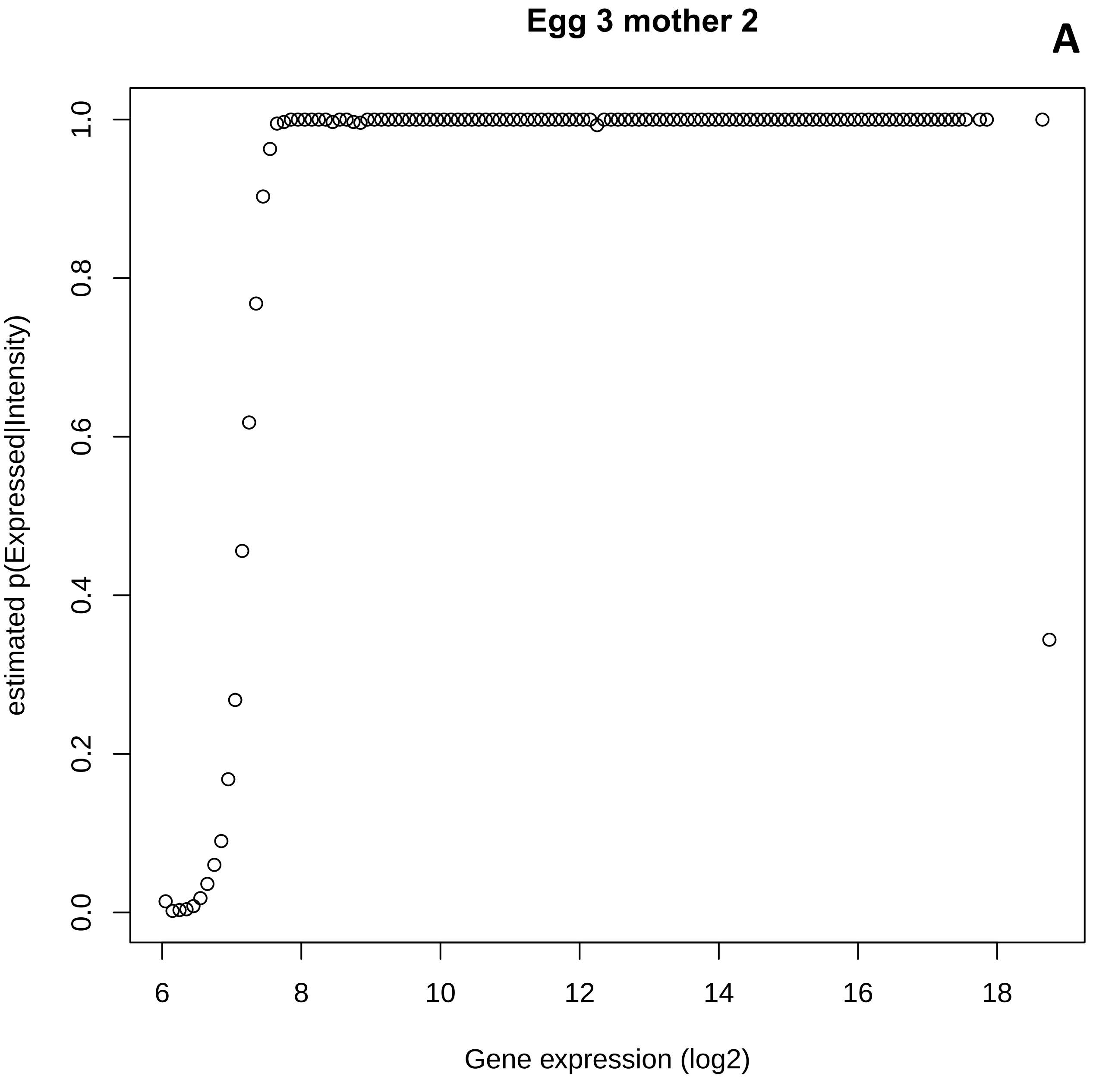
Egg 3 mother 2
A
estimated p(Expressed|Intensity)
0.0
0.2
0.4
0.6
0.8
1.0
6
8
10
12
14
16
18
Gene expression (log2)

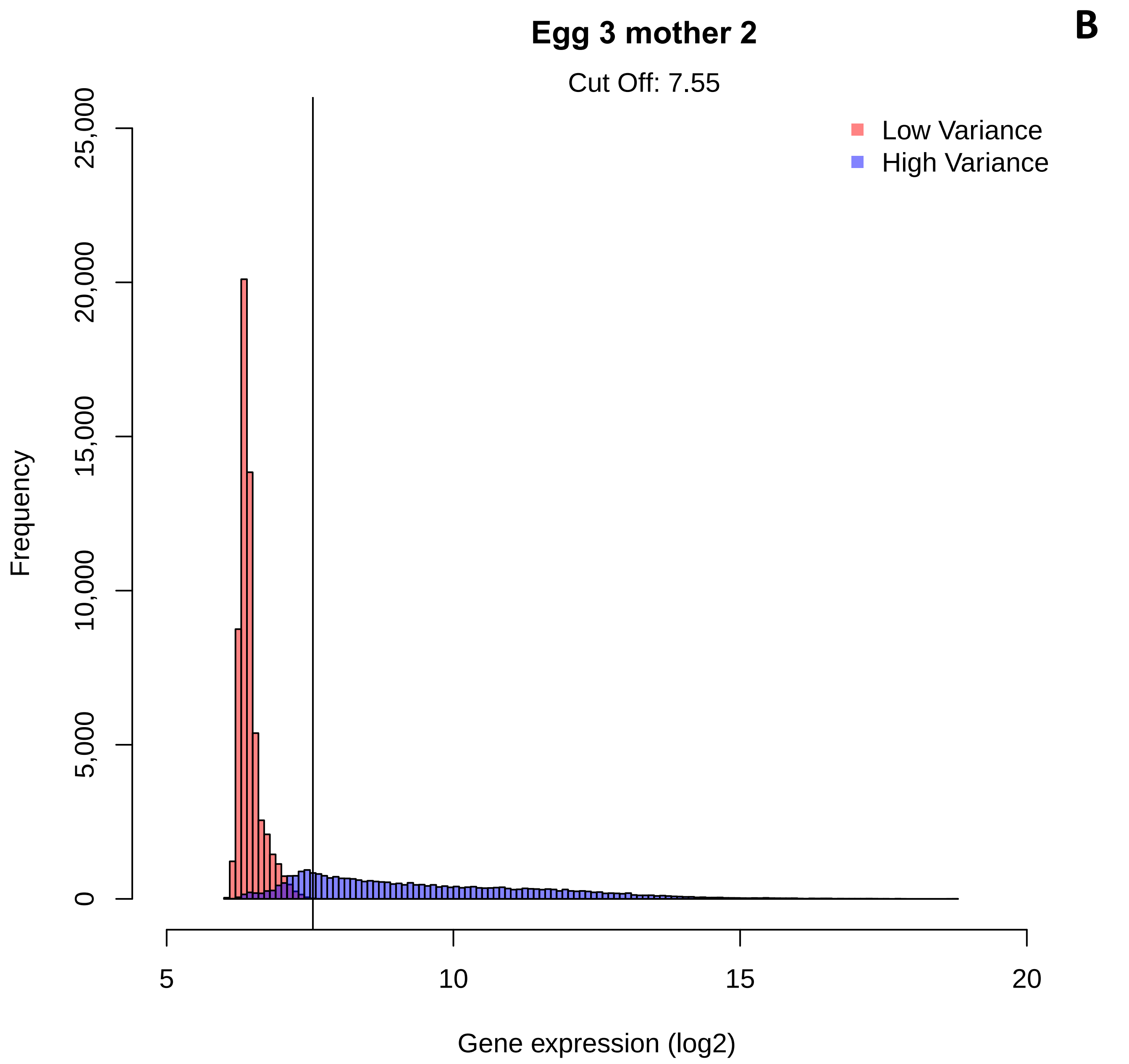

B
Egg 3 mother 2
Cut Off: 7.55
Low Variance
High Variance
Frequency
0
5,000
10,000
15,000
20,000
25,000
5
10
15
20
Gene expression (log2)

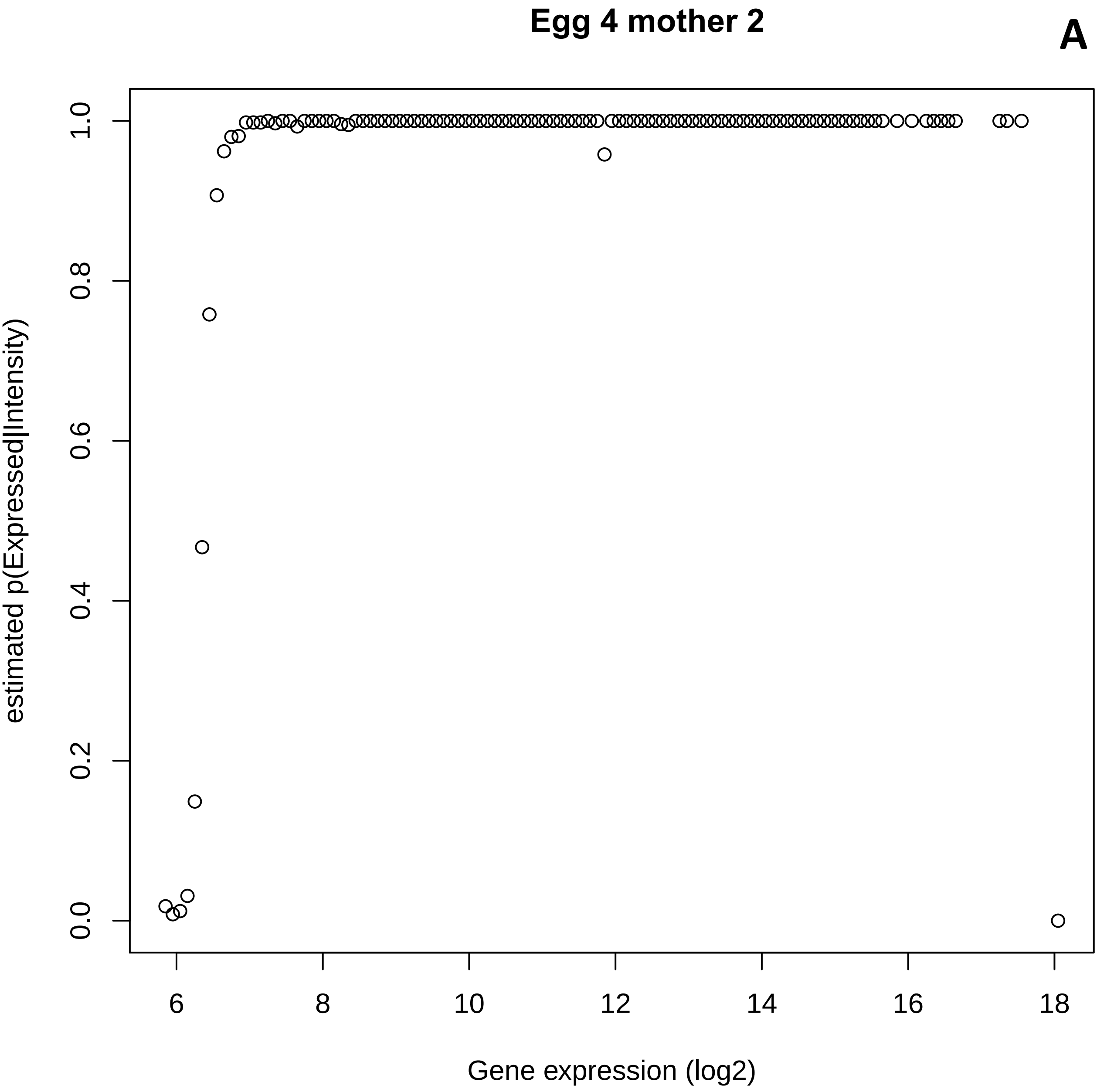

Egg 4 mother 2
A
estimated p(Expressed|Intensity)
0.0
0.2
0.4
0.6
0.8
1.0
6
8
10
12
14
16
18
Gene expression (log2)

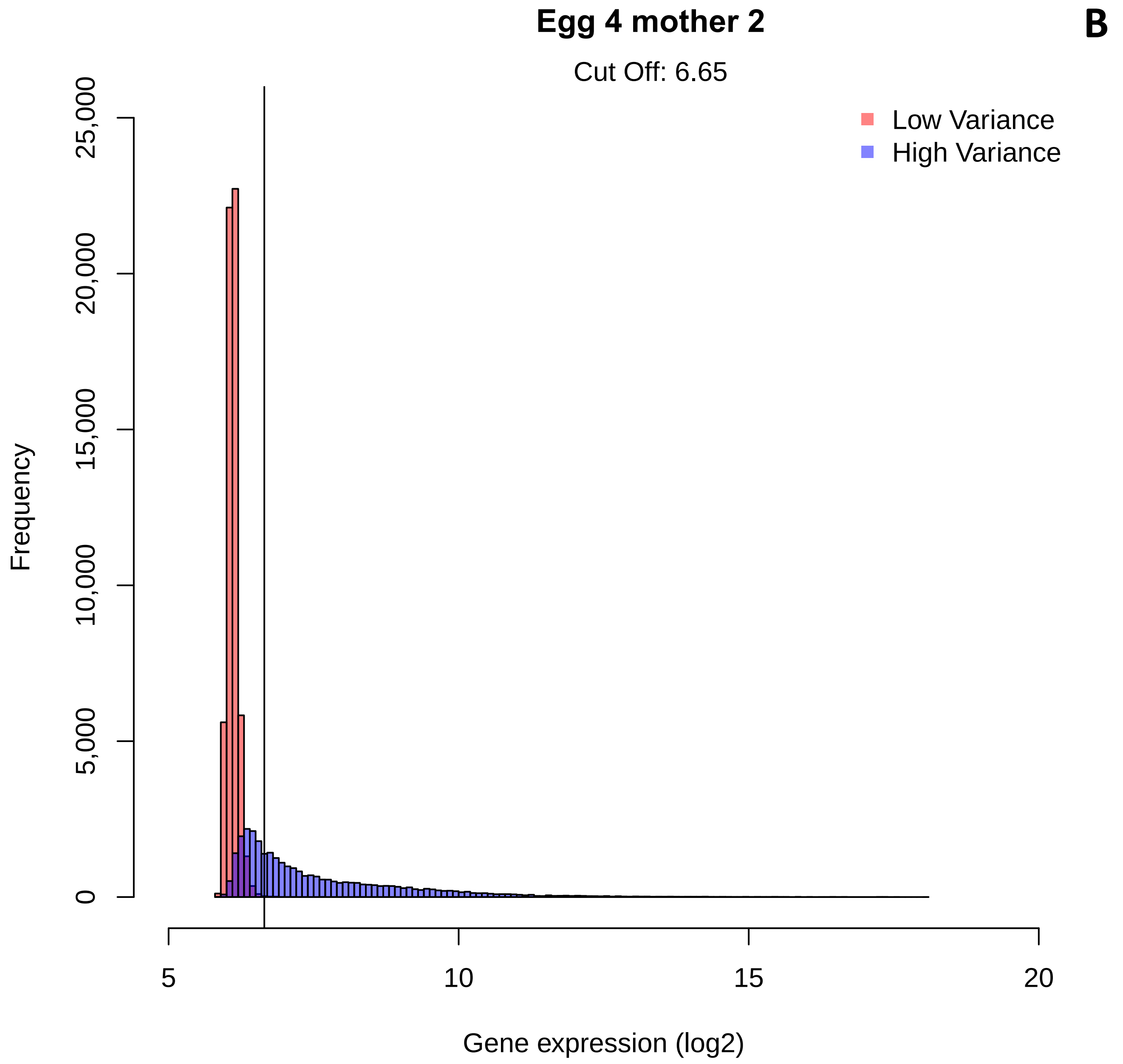

Egg 4 mother 2
B
Cut Off: 6.65
Low Variance
High Variance
Frequency
0
5,000
10,000
15,000
20,000
25,000
5
10
15
20
Gene expression (log2)

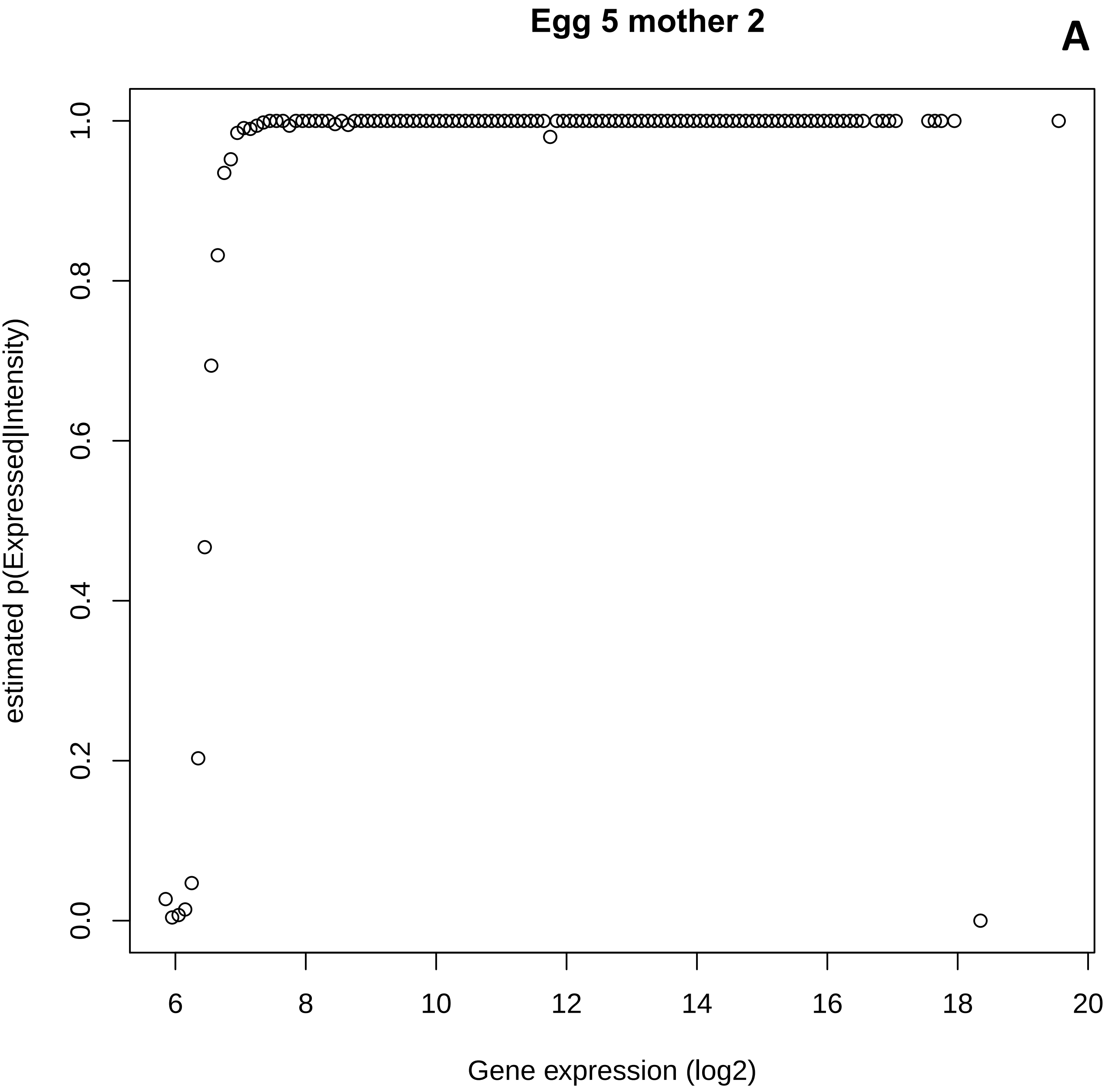
Egg 5 mother 2
A
estimated p(Expressed|Intensity)
0.0
0.2
0.4
0.6
0.8
1.0
6
8
10
12
14
16
18
20
Gene expression (log2)

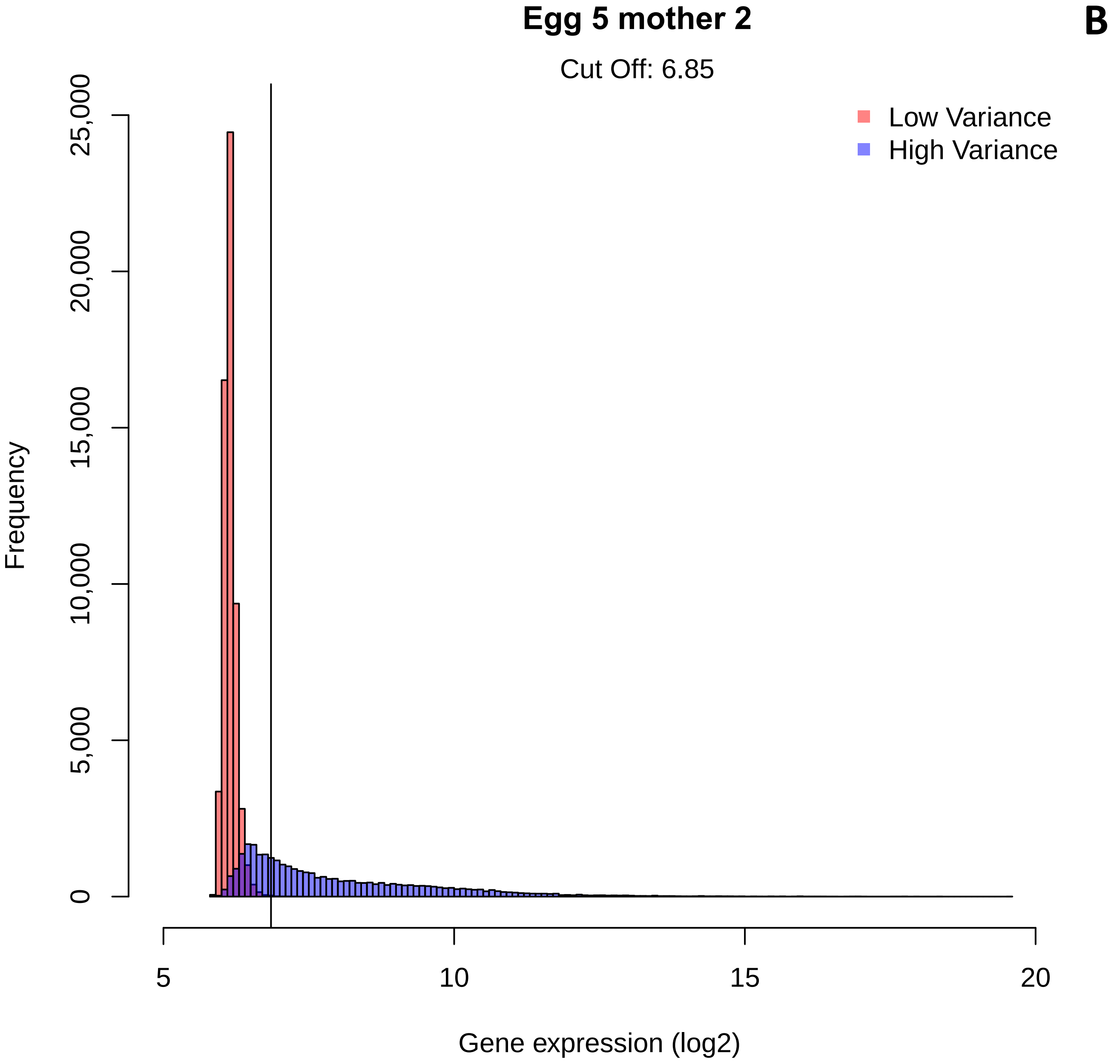
Egg 5 mother 2
Cut Off: 6.85
B
Low Variance
High Variance
Frequency
25,000
20,000
15,000
10,000
5,000
0
5
10
15
20
Gene expression (log2)

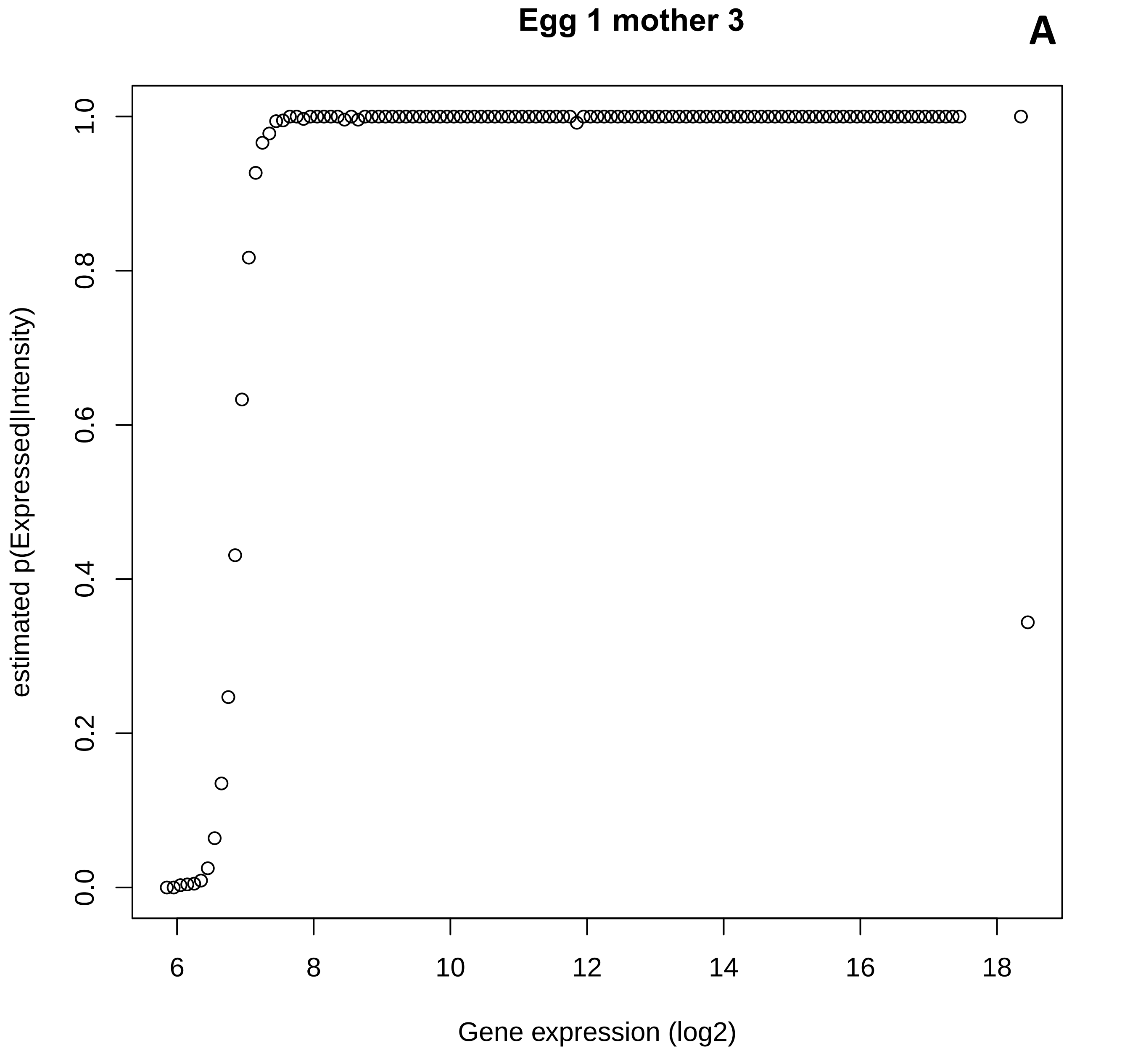

Egg 1 mother 3
A
estimated p(Expressed|Intensity)
0.0
0.2
0.4
0.6
0.8
1.0
6
8
10
12
14
16
18
Gene expression (log2)

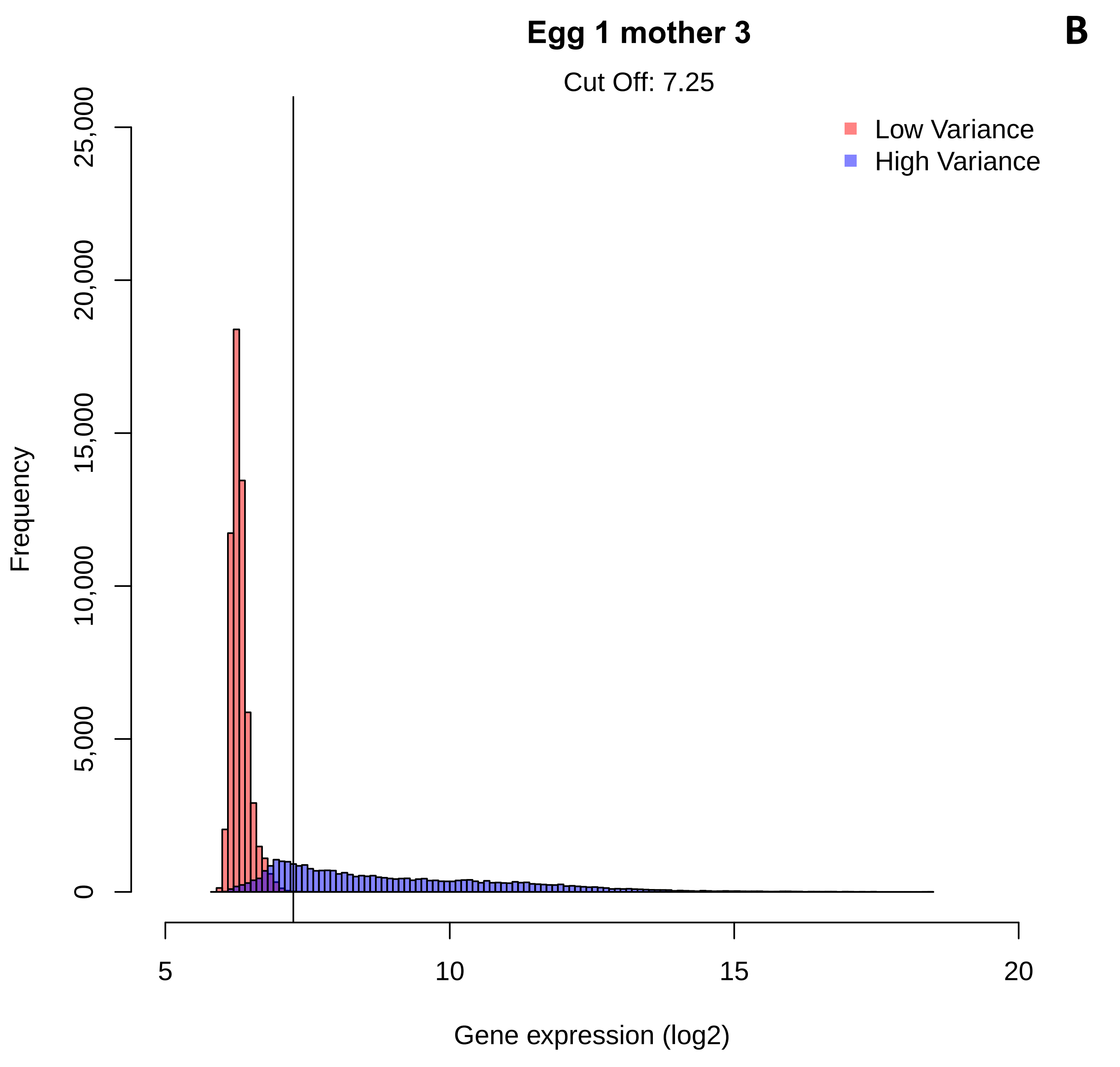

B
Egg 1 mother 3
Cut Off: 7.25
Low Variance
High Variance
Frequency
0
5,000
10,000
15,000
20,000
25,000
5
10
15
20
Gene expression (log2)

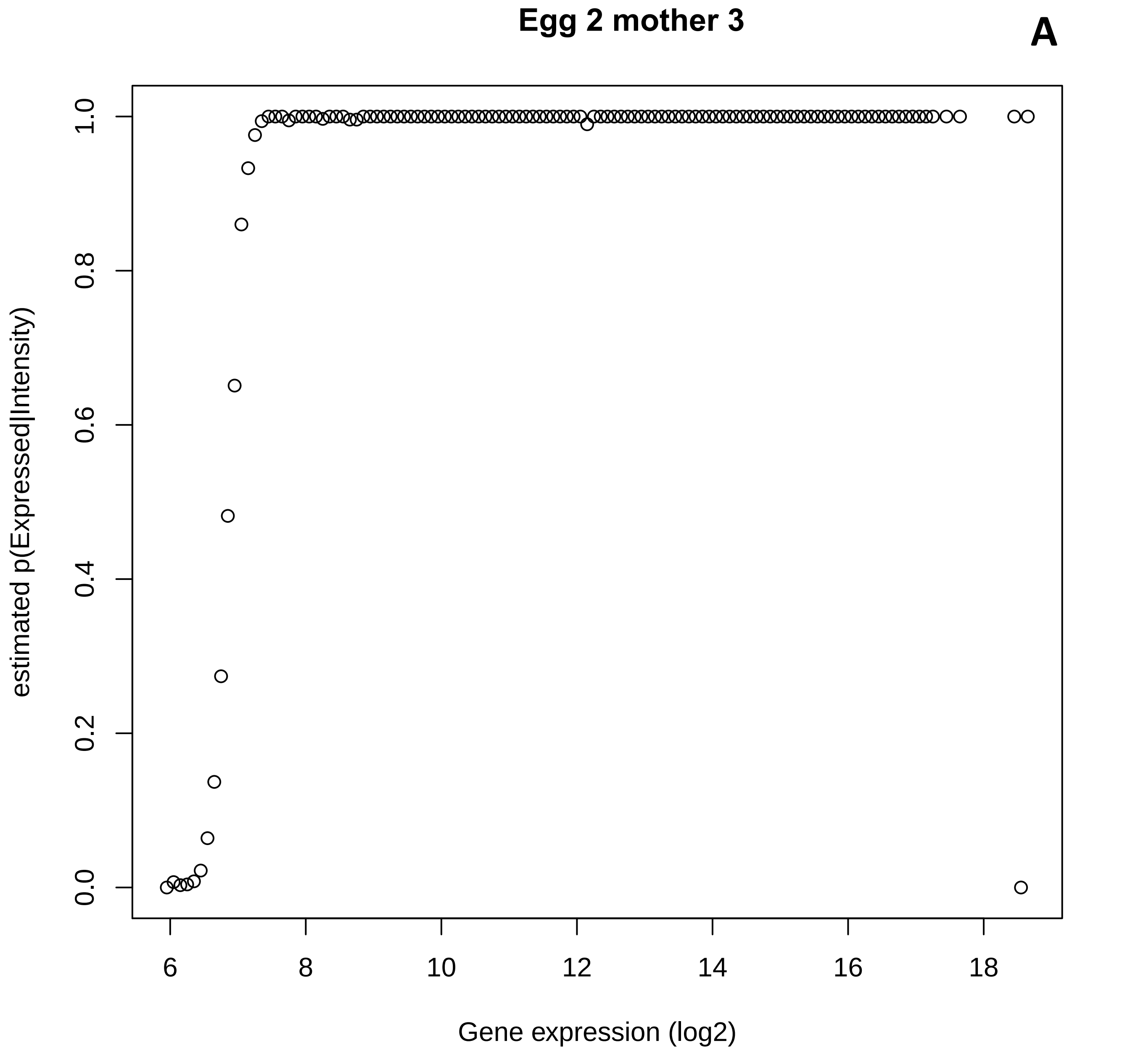
Egg 2 mother 3
A
estimated p(Expressed|Intensity)
0.0
0.2
0.4
0.6
0.8
1.0
6
8
10
12
14
16
18
Gene expression (log2)

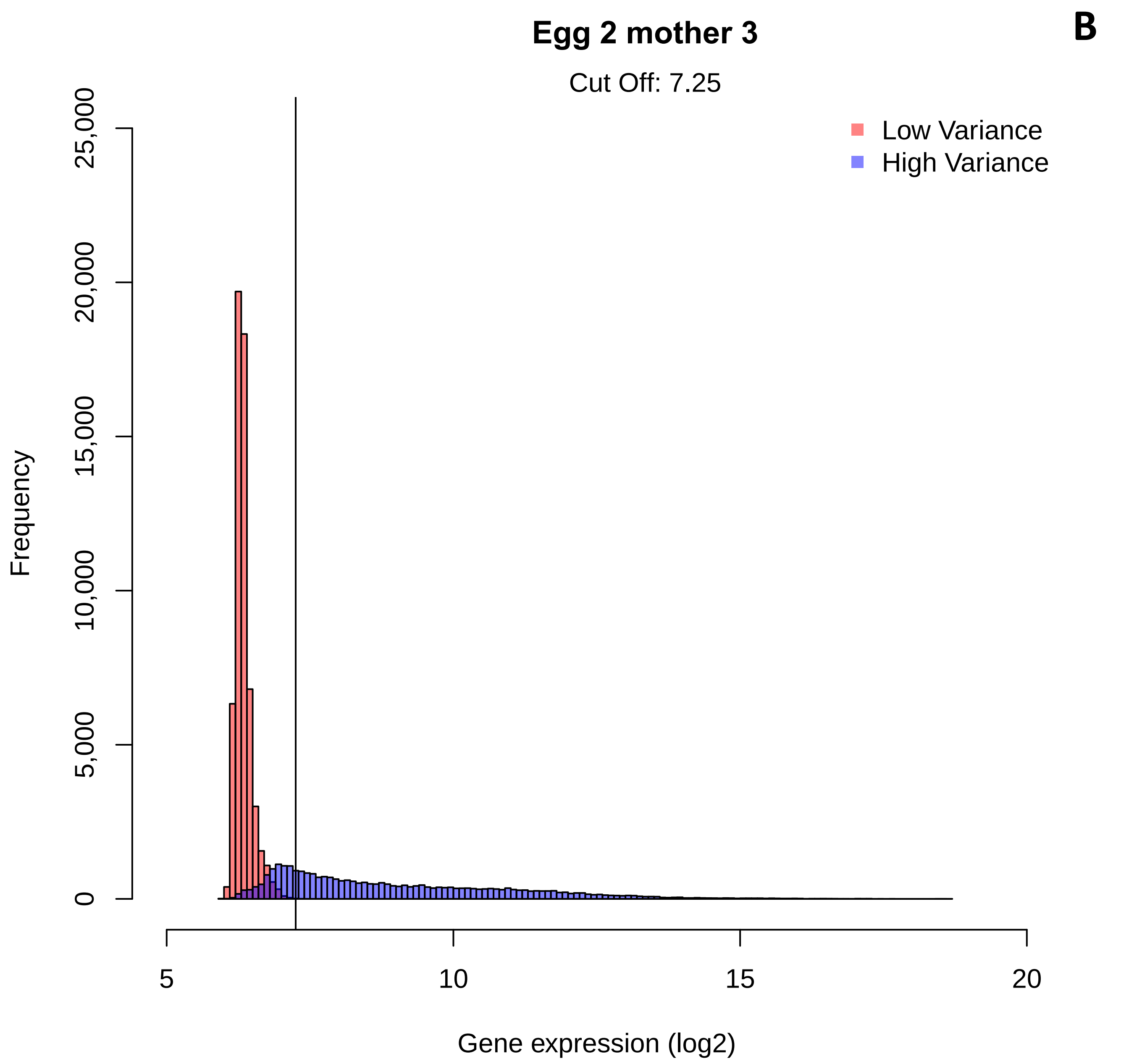

B
Egg 2 mother 3
Cut Off: 7.25
Low Variance
High Variance
Frequency
0
5,000
10,000
15,000
20,000
25,000
5
10
15
20
Gene expression (log2)

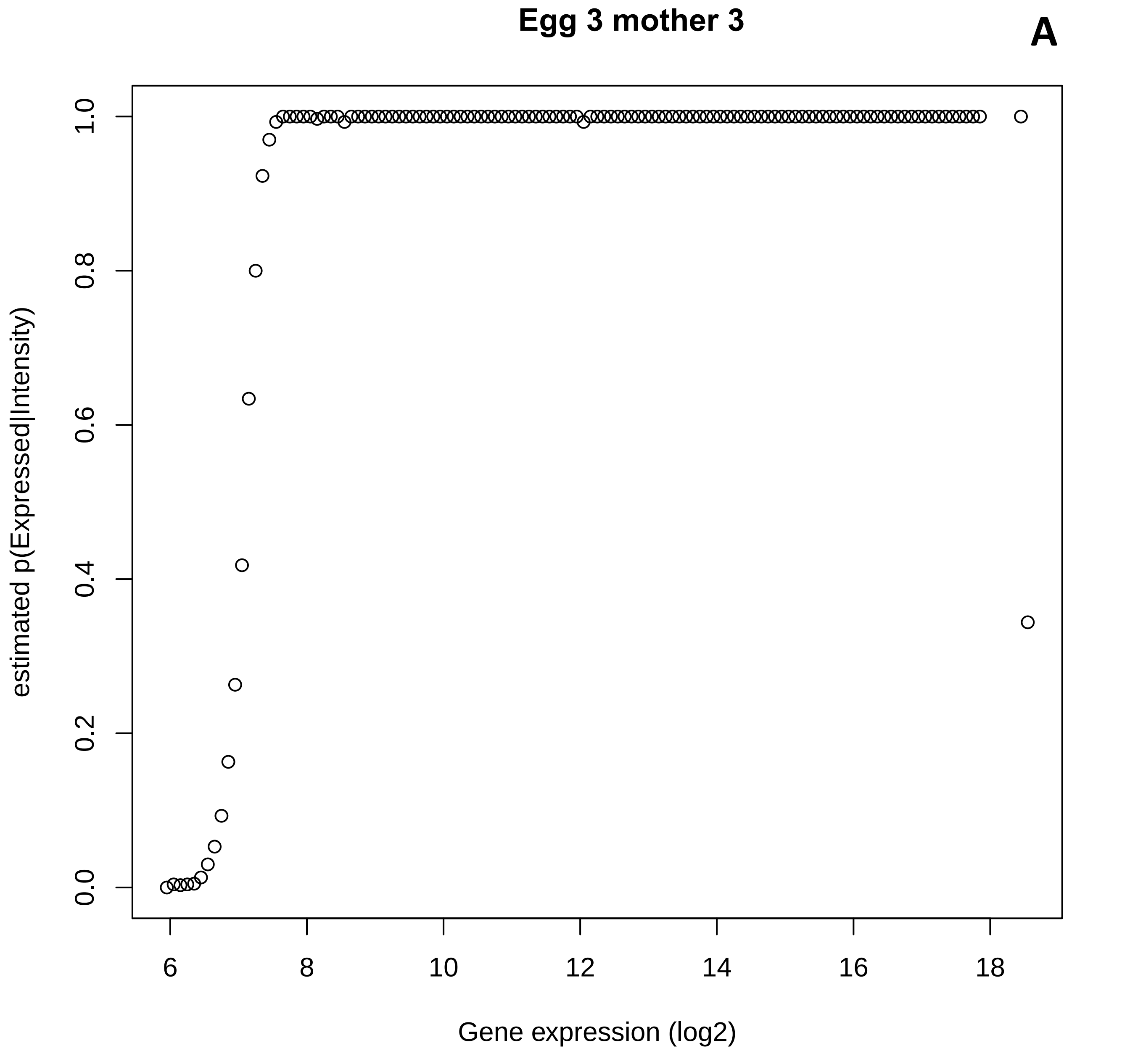

Egg 3 mother 3
A
estimated p(Expressed|Intensity)
0.0
0.2
0.4
0.6
0.8
1.0
6
8
10
12
14
16
18
Gene expression (log2)

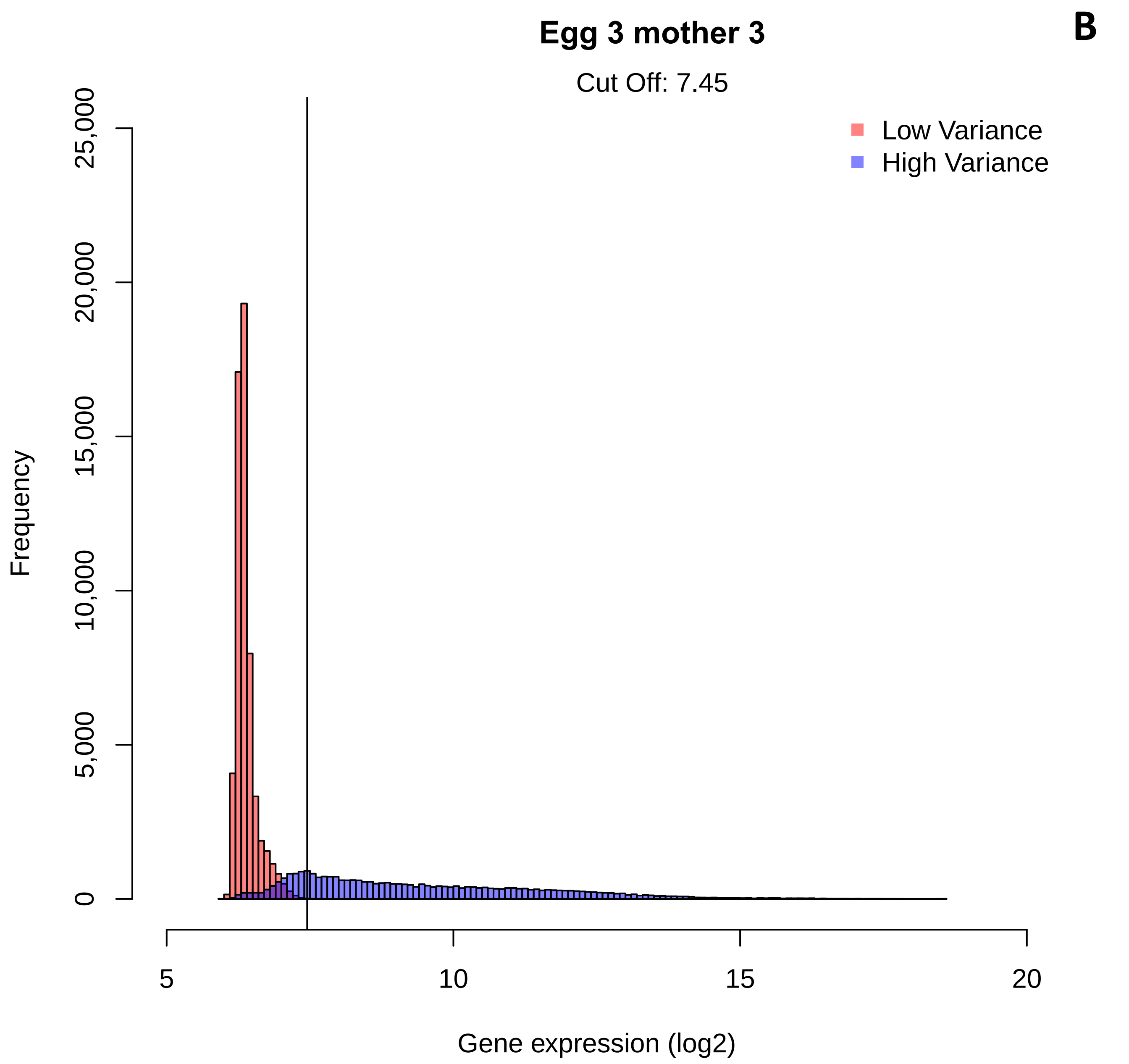
B
Egg 3 mother 3
Cut Off: 7.45
Low Variance
High Variance
Frequency
0
5,000
10,000
15,000
20,000
25,000
5
10
15
20
Gene expression (log2)

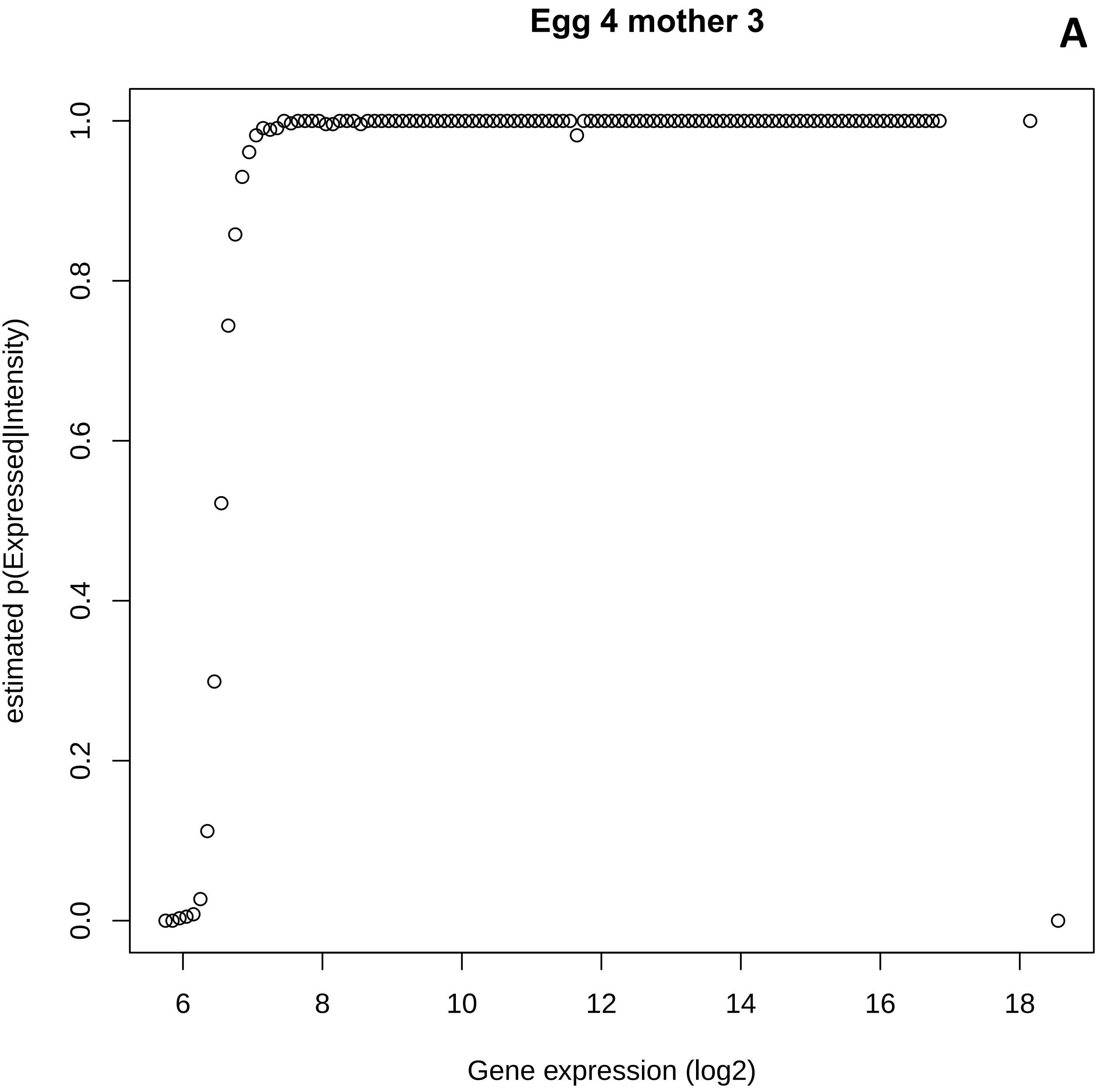

Egg 4 mother 3
A
estimated p(Expressed|Intensity)
0.0
0.2
0.4
0.6
0.8
1.0
6
8
10
12
14
16
18
Gene expression (log2)

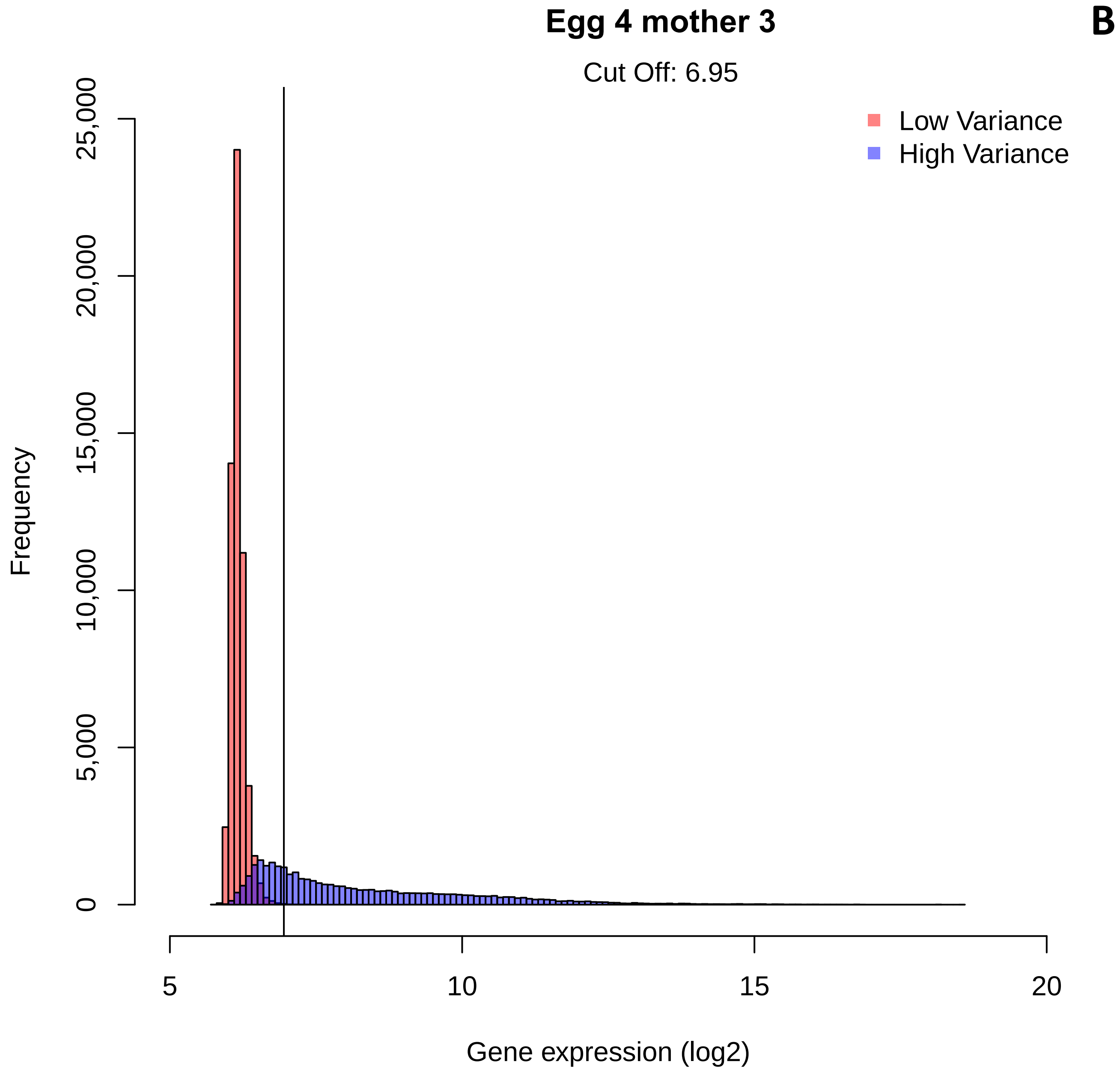

Egg 4 mother 3
B
Cut Off: 6.95
Low Variance
High Variance
Frequency
25,000
20,000
15,000
10,000
5,000
0
5
10
15
20
Gene expression (log2)

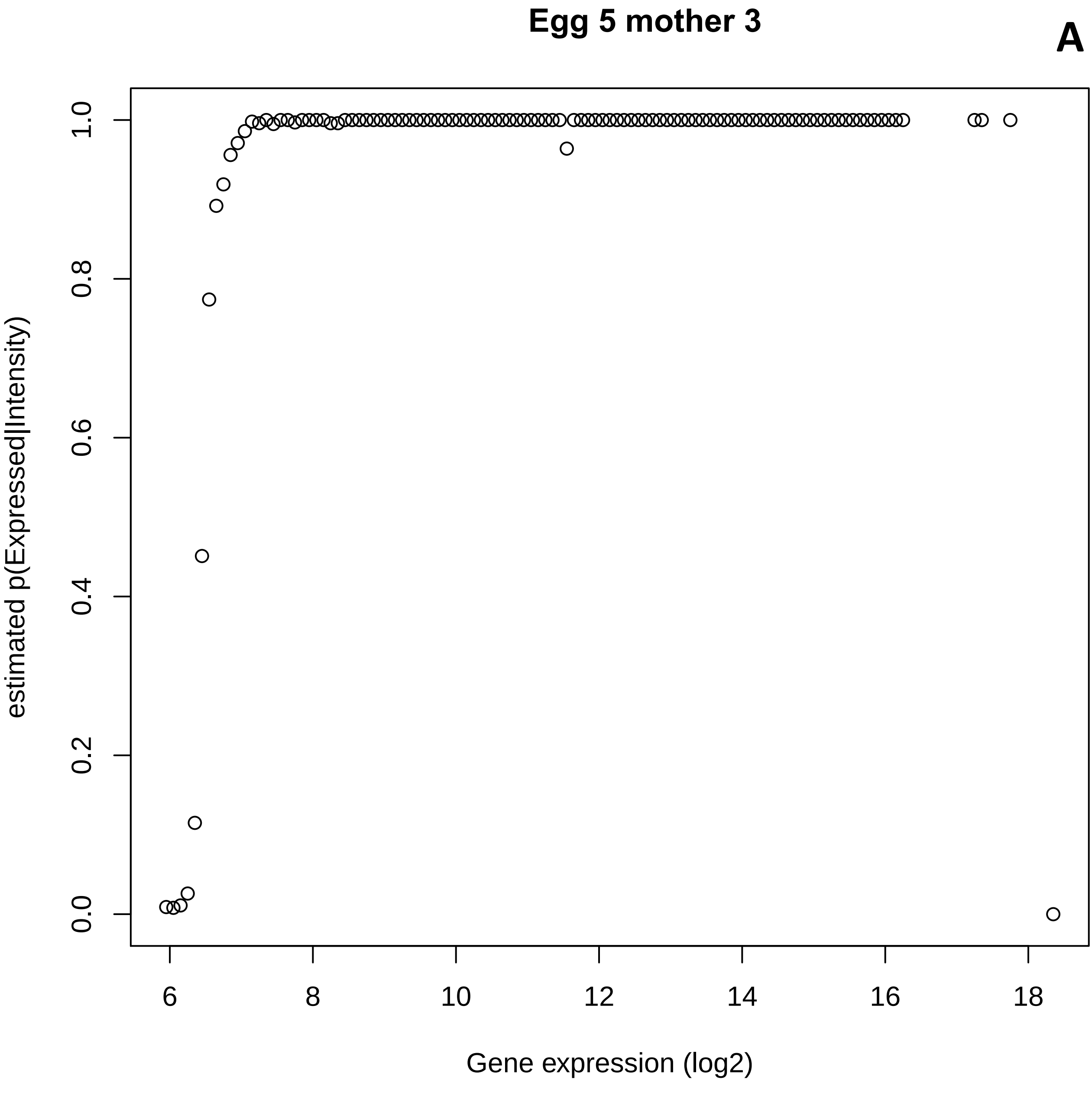

Egg 5 mother 3
A
estimated p(Expressed|Intensity)
0.0
0.2
0.4
0.6
0.8
1.0
6
8
10
12
14
16
18
Gene expression (log2)

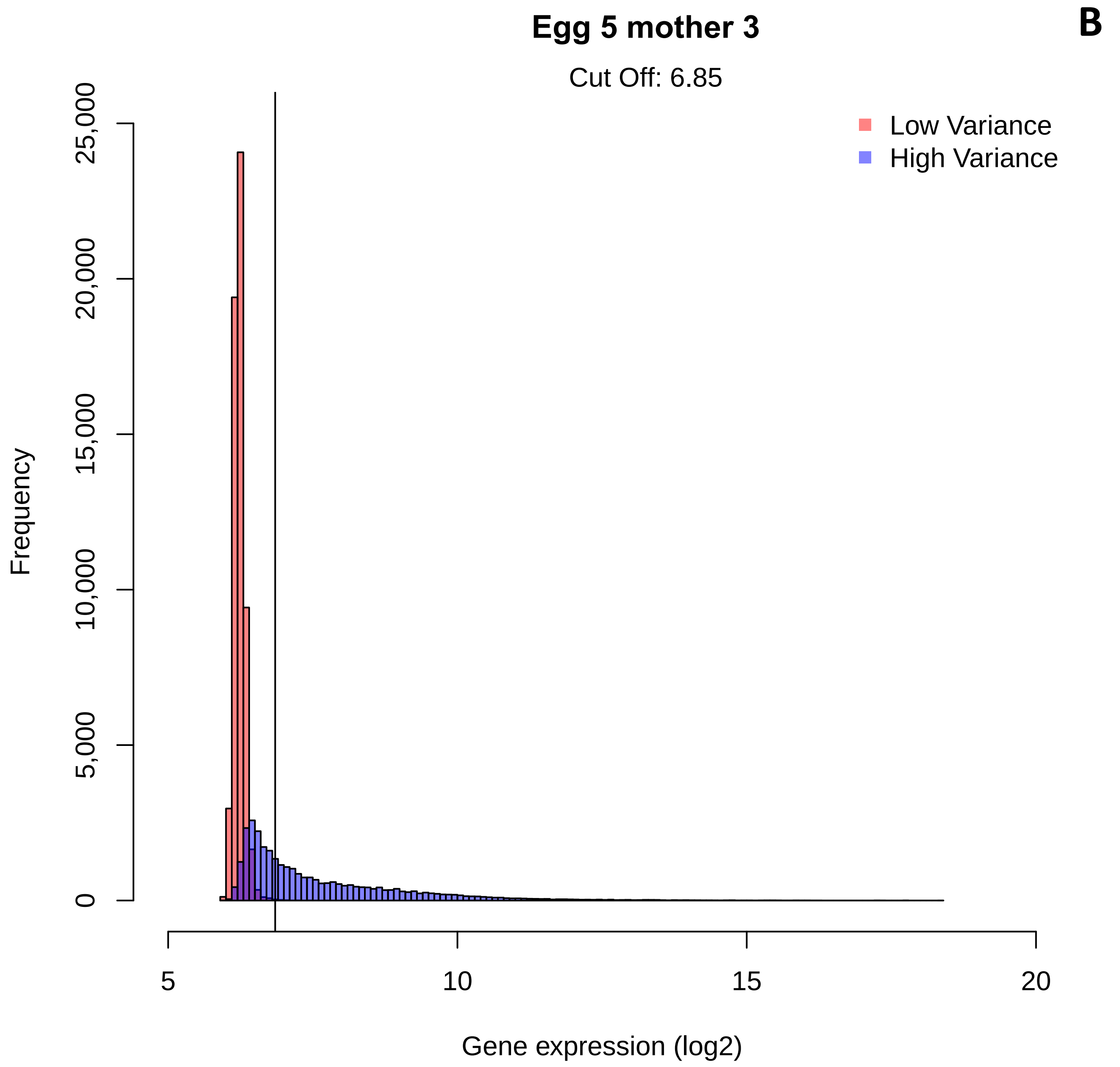

B
Egg 5 mother 3
Cut Off: 6.85
Low Variance
High Variance
Frequency
0
5,000
10,000
15,000
20,000
25,000
5
10
15
20
Gene expression (log2)

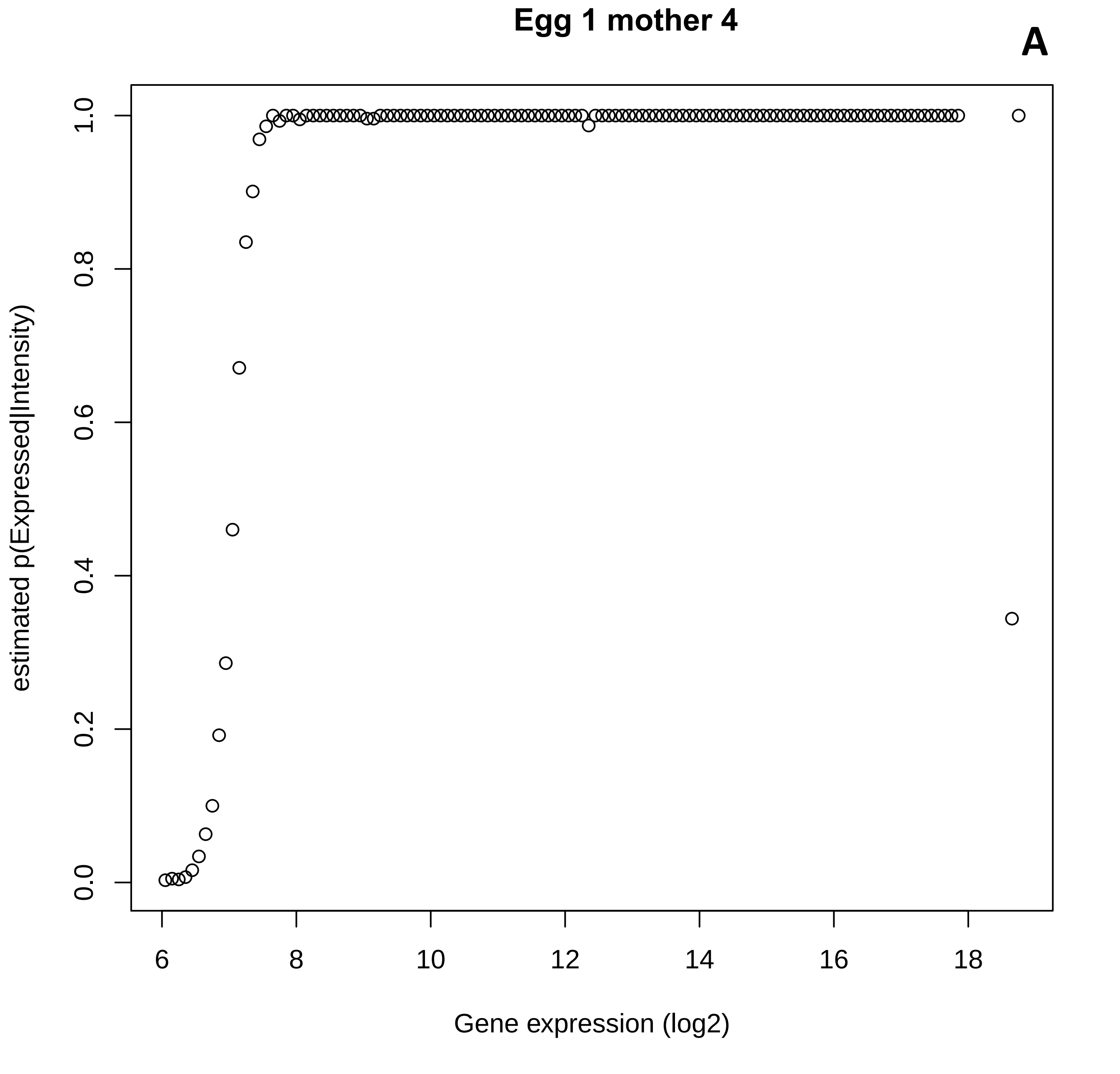
Egg 1 mother 4
A
estimated p(Expressed|Intensity)
0.0
0.2
0.4
0.6
0.8
1.0
6
8
10
12
14
16
18
Gene expression (log2)

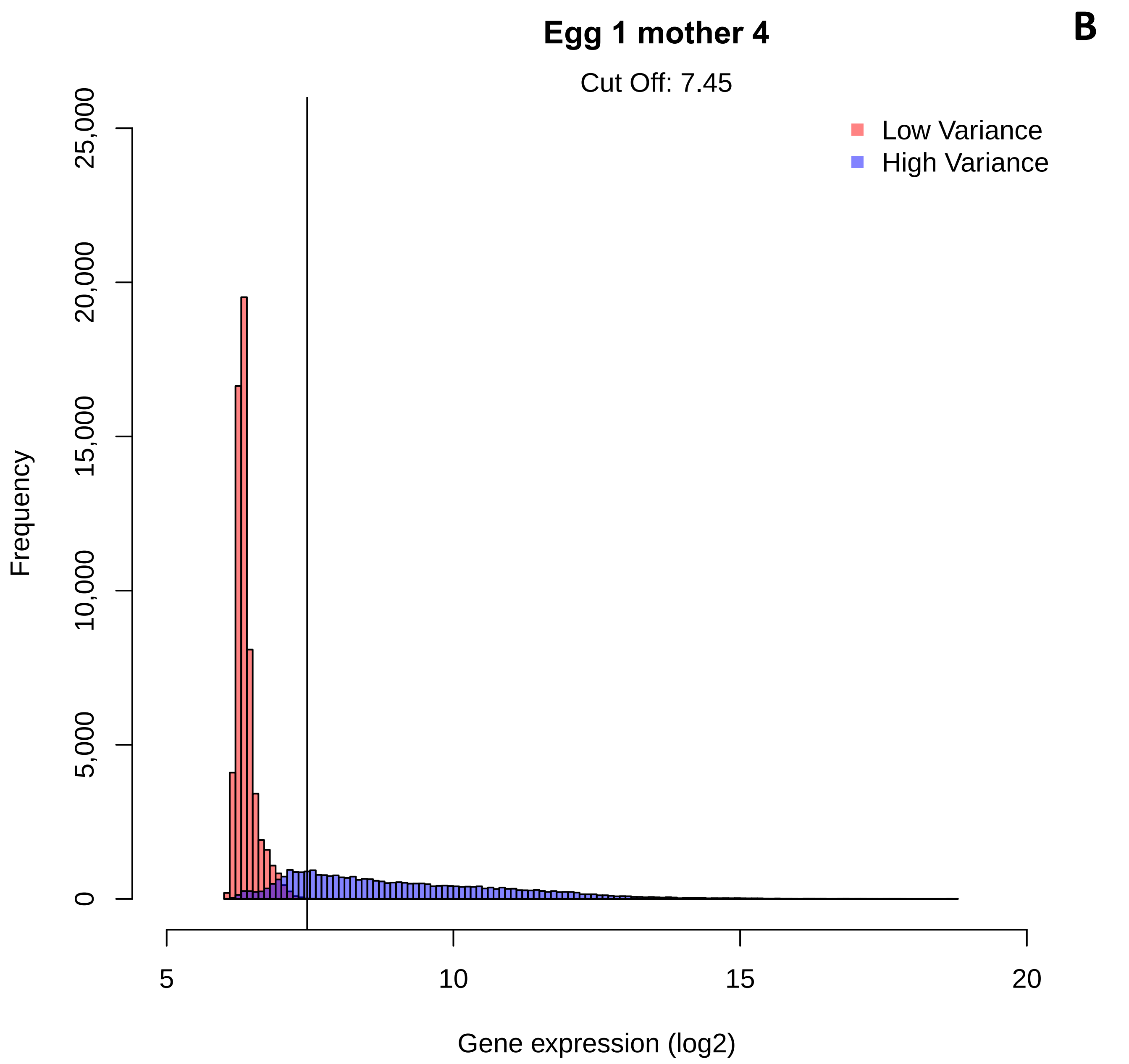

B
Egg 1 mother 4
Cut Off: 7.45
Low Variance
High Variance
Frequency
25,000
20,000
15,000
10,000
5,000
0
5
10
15
20
Gene expression (log2)

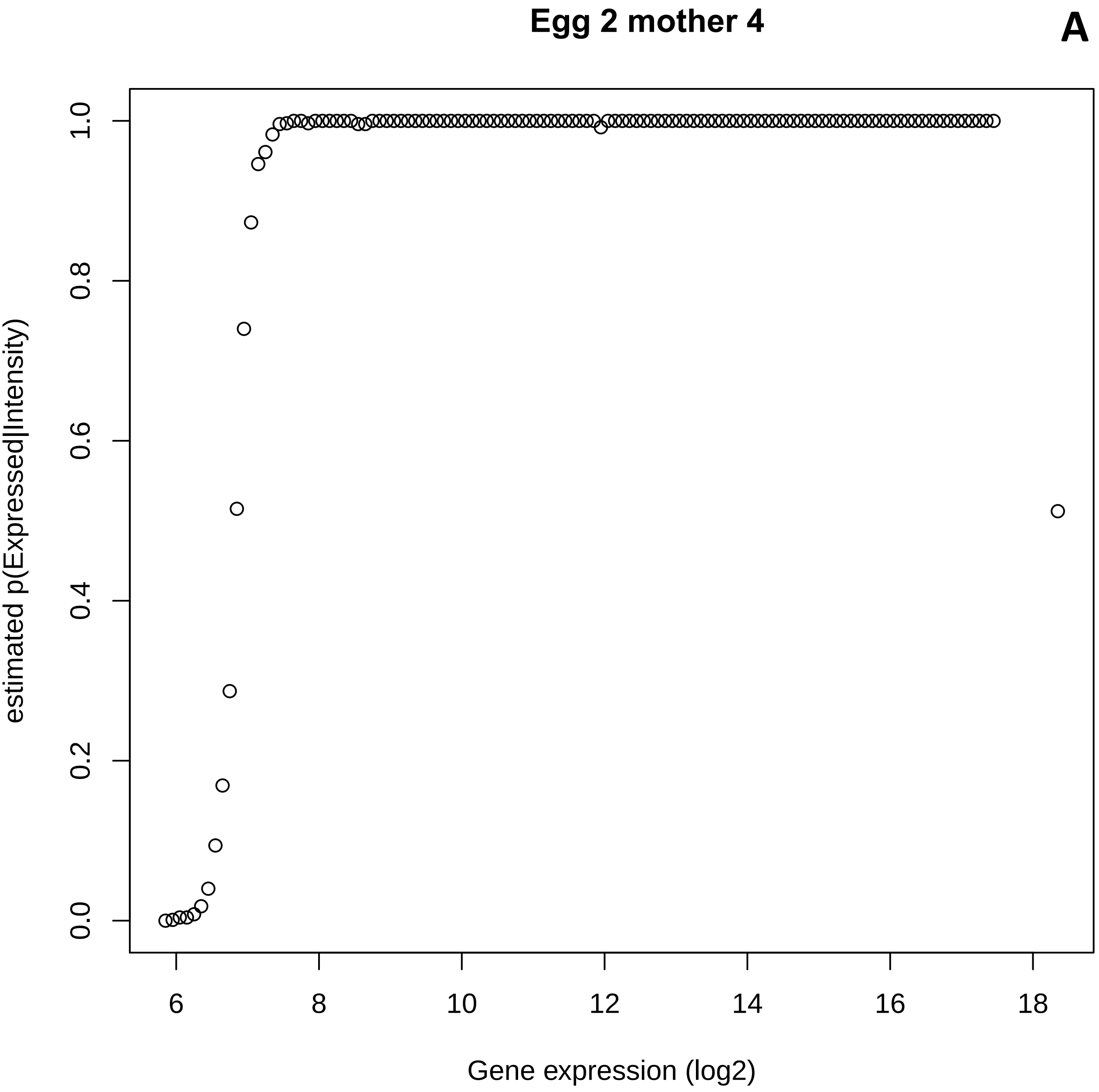
Egg 2 mother 4
A
estimated p(Expressed|Intensity)
0.0
0.2
0.4
0.6
0.8
1.0
6
8
10
12
14
16
18
Gene expression (log2)

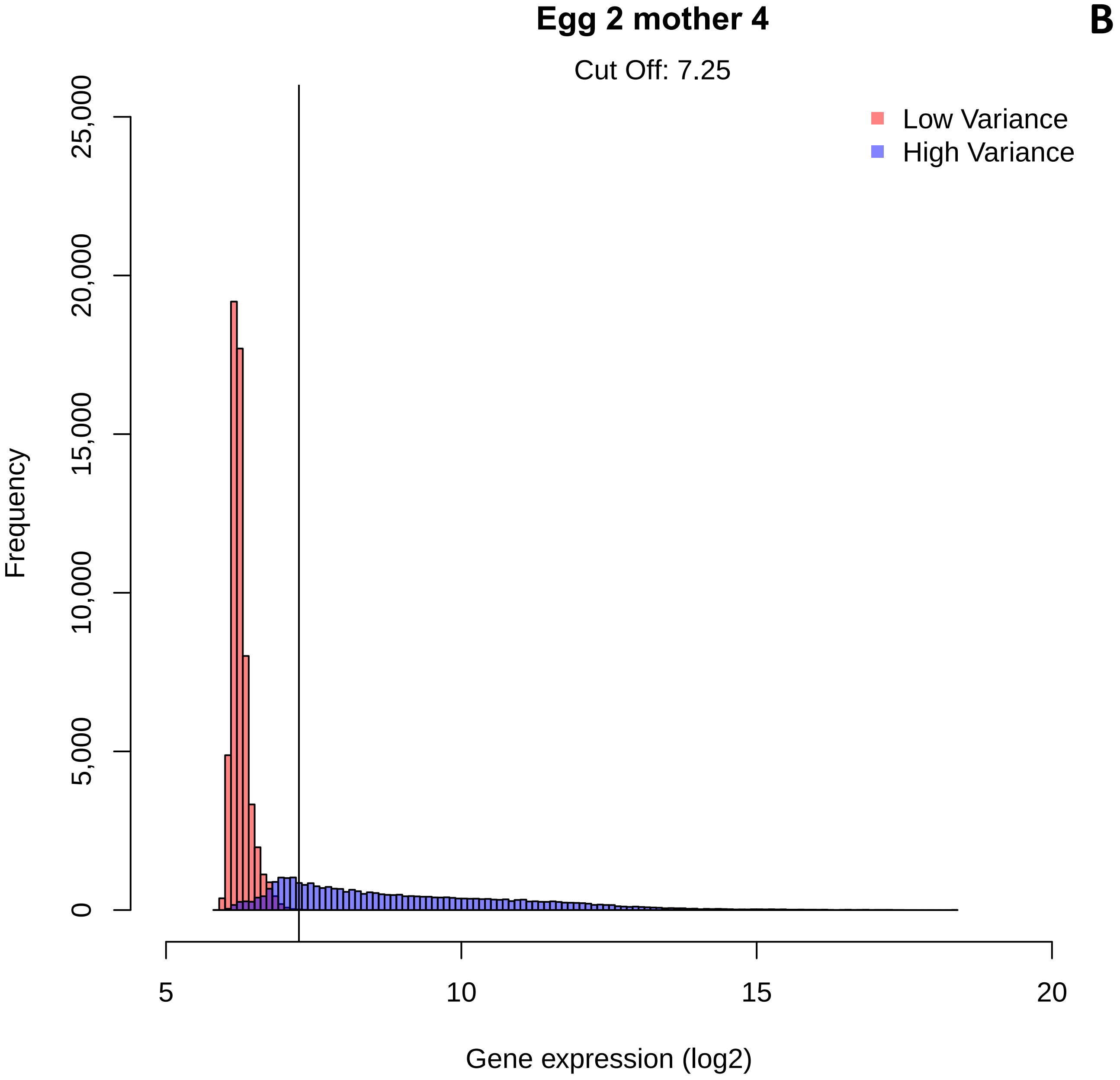
Egg 2 mother 4
B
Cut Off: 7.25
Low Variance
High Variance
Frequency
0
5,000
10,000
15,000
20,000
25,000
5
10
15
20
Gene expression (log2)

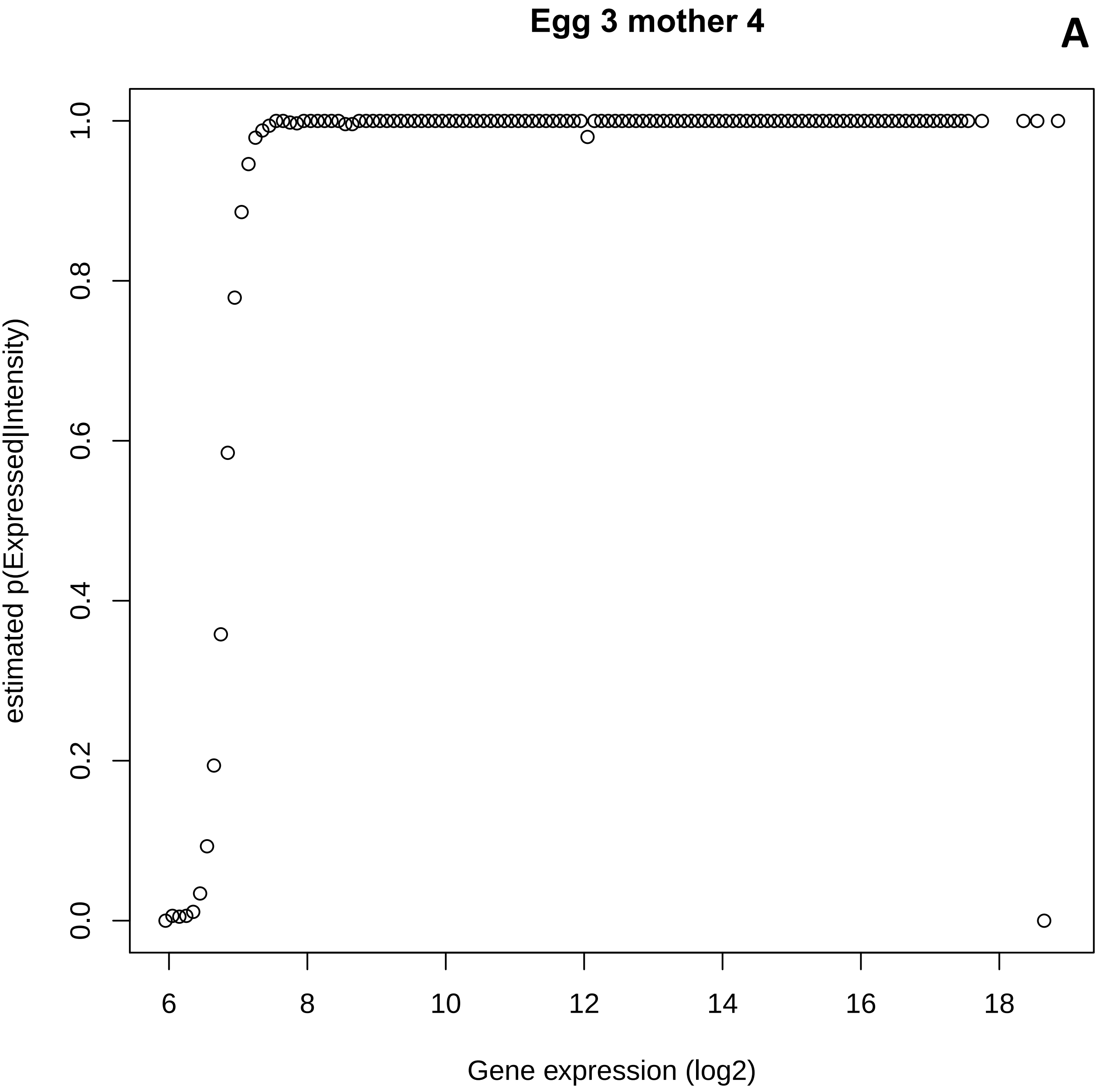

Egg 3 mother 4
A
estimated p(Expressed|Intensity)
0.0
0.2
0.4
0.6
0.8
1.0
6
8
10
12
14
16
18
Gene expression (log2)

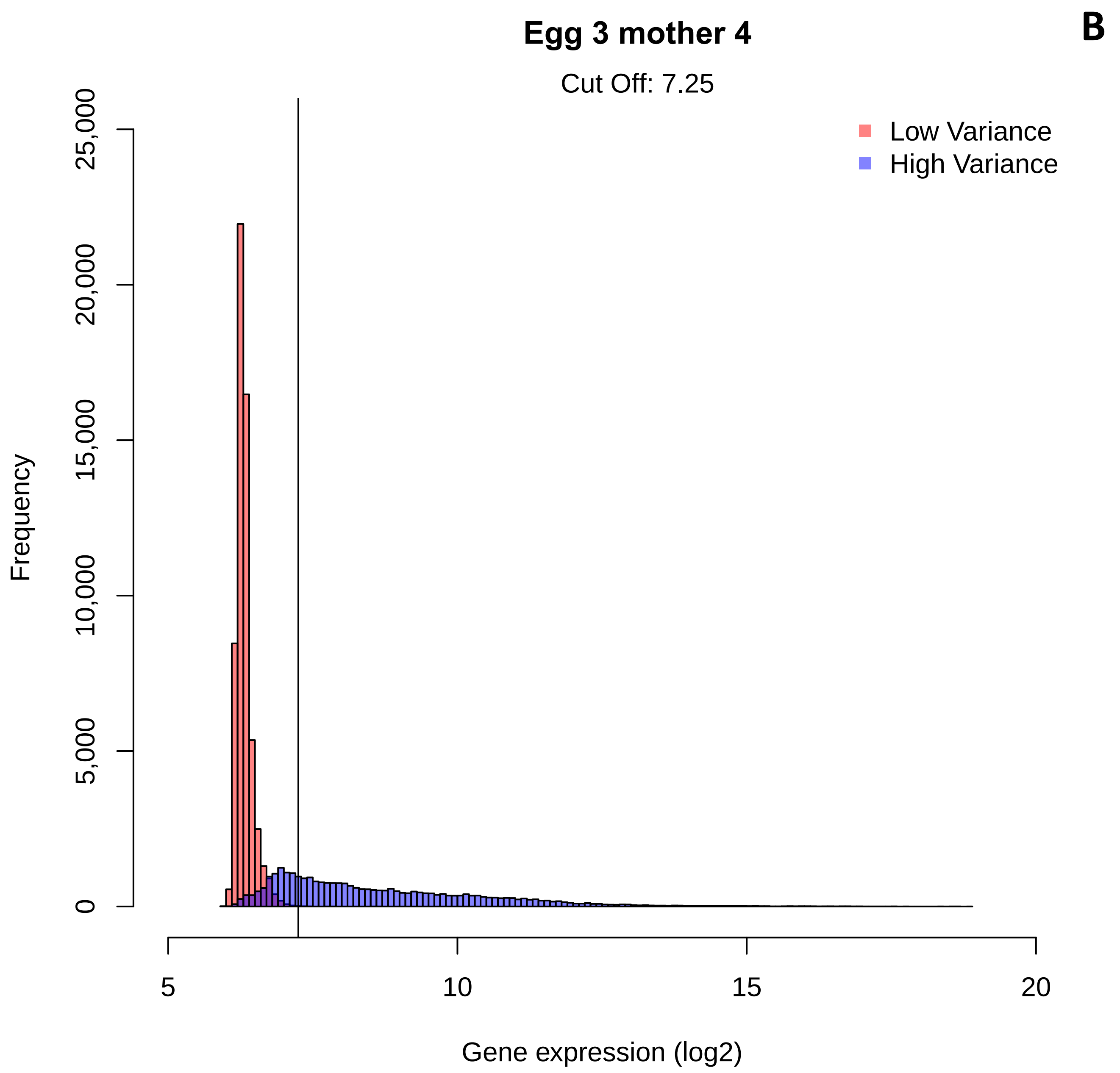
B
Egg 3 mother 4
Cut Off: 7.25
Low Variance
High Variance
Frequency
0
5,000
10,000
15,000
20,000
25,000
5
10
15
20
Gene expression (log2)

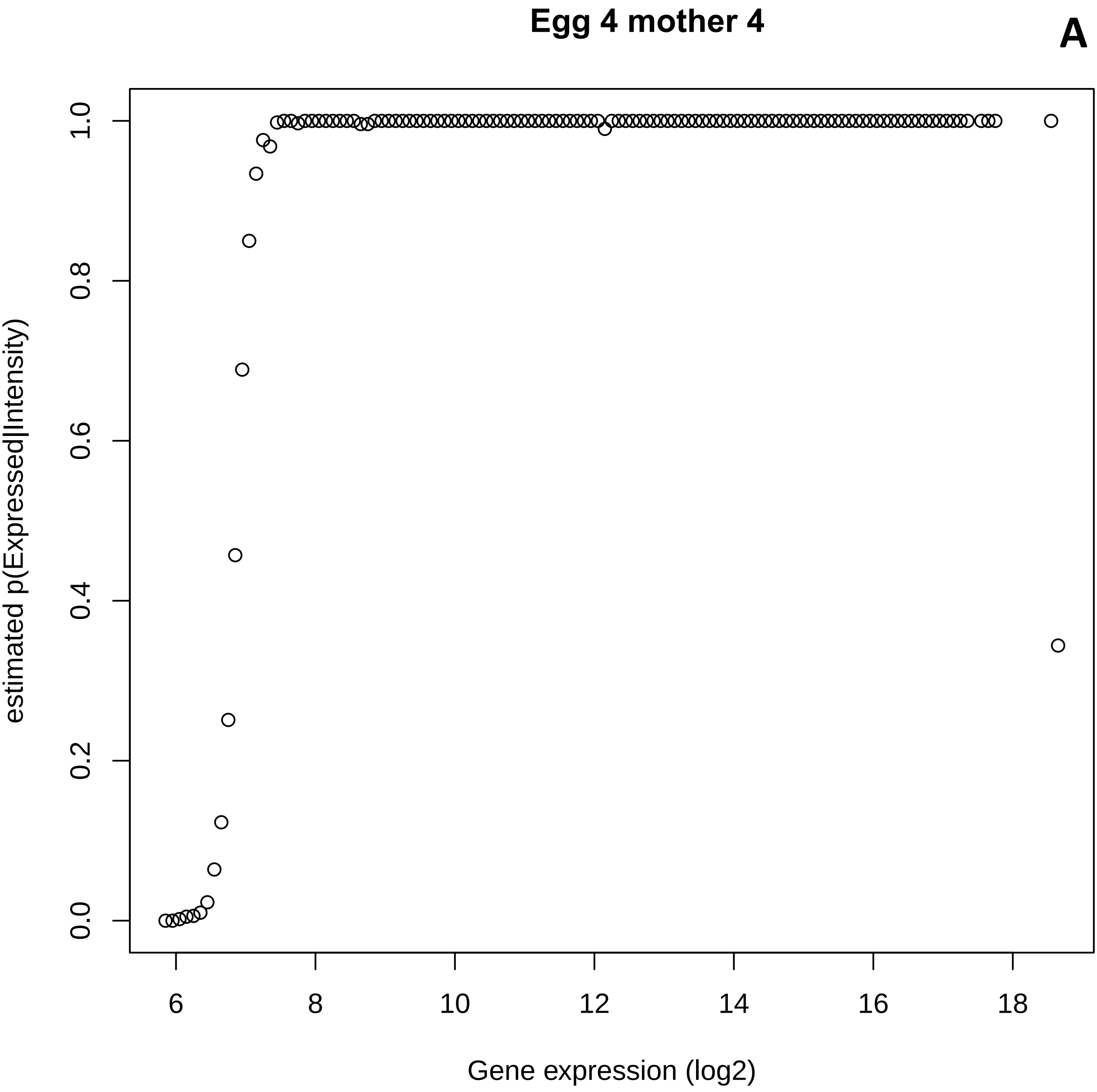

Egg 4 mother 4
A
estimated p(Expressed|Intensity)
0.0
0.2
0.4
0.6
0.8
1.0
6
8
10
12
14
16
18
Gene expression (log2)

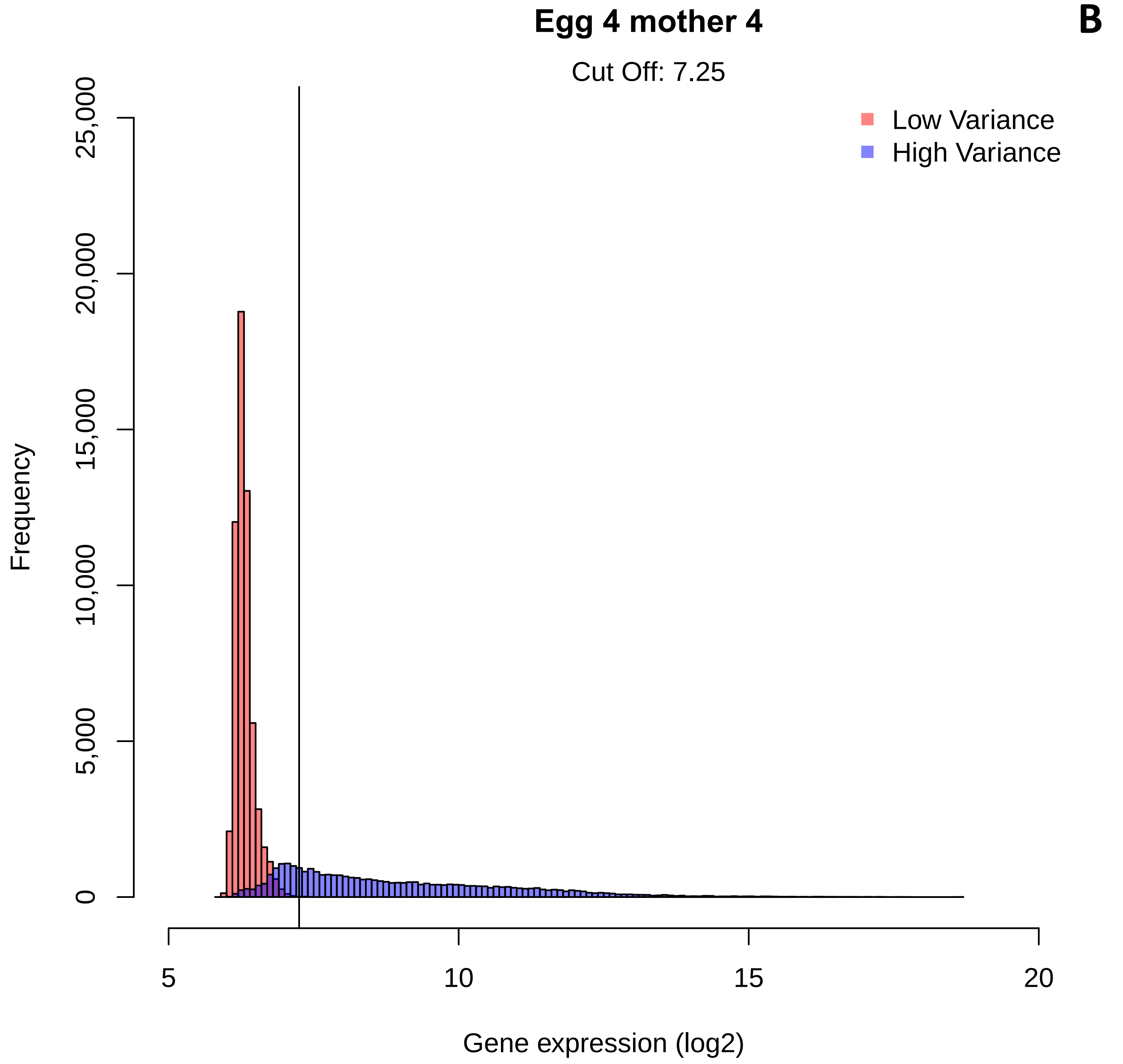

Egg 4 mother 4
Cut Off: 7.25
B
Low Variance
High Variance
Frequency
0
5,000
10,000
15,000
20,000
25,000
5
10
15
20
Gene expression (log2)

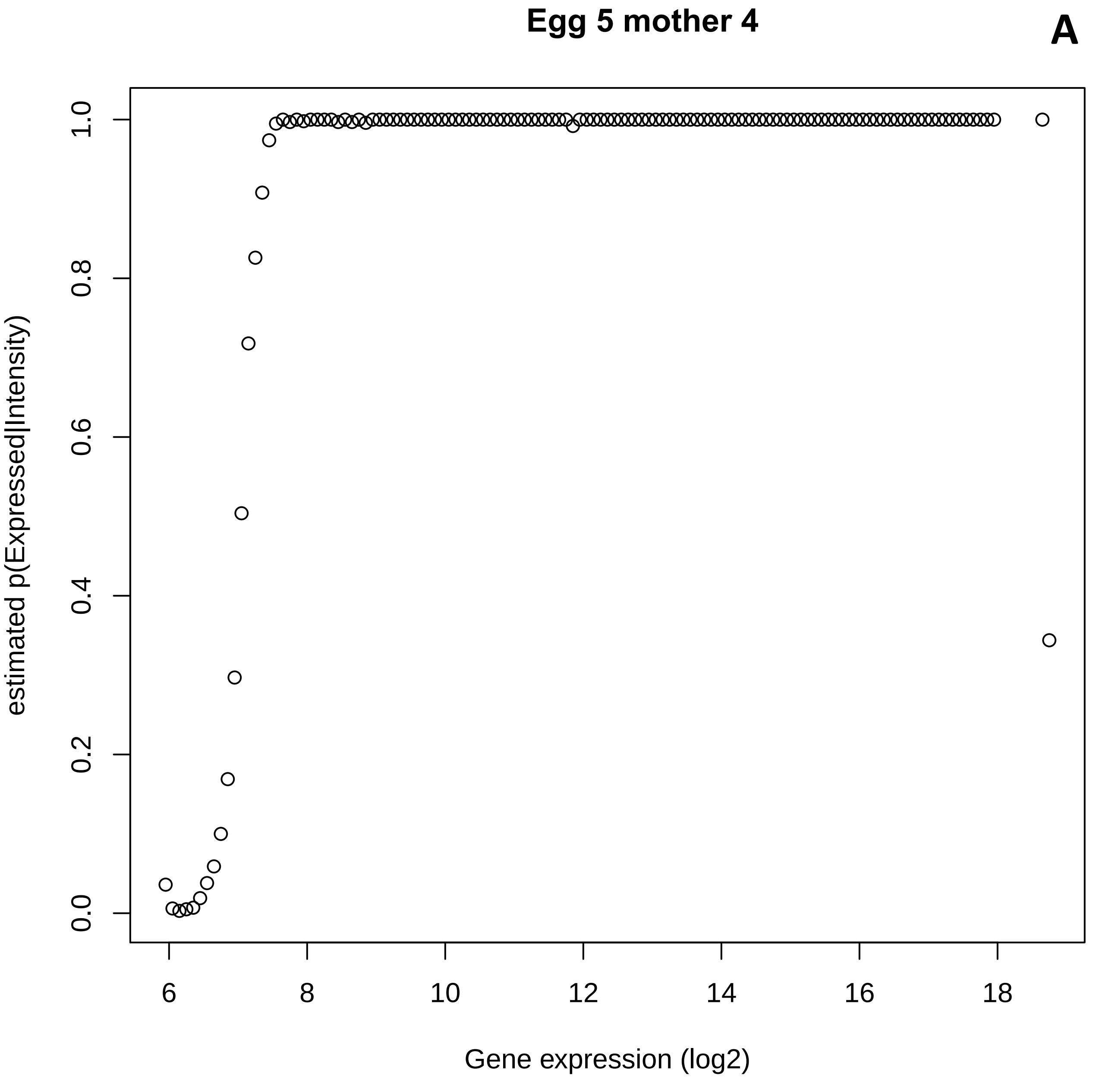

Egg 5 mother 4
A
estimated p(Expressed|Intensity)
0.0
0.2
0.4
0.6
0.8
1.0
6
8
10
12
14
16
18
Gene expression (log2)

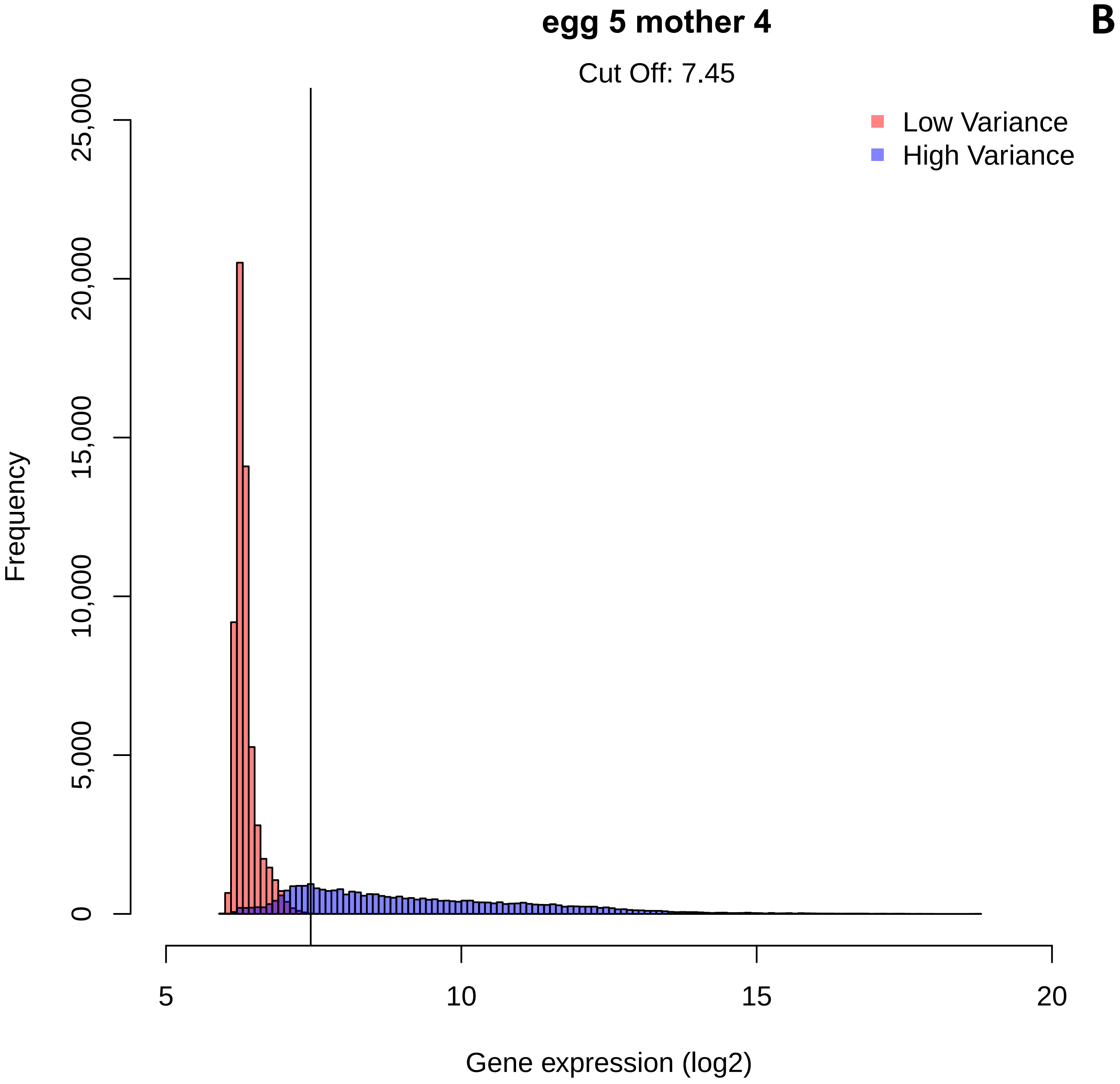
B
egg 5 mother 4
Cut Off: 7.45
Low Variance
High Variance
Frequency
0
5,000
10,000
15,000
20,000
25,000
5
10
15
20
Gene expression (log2)

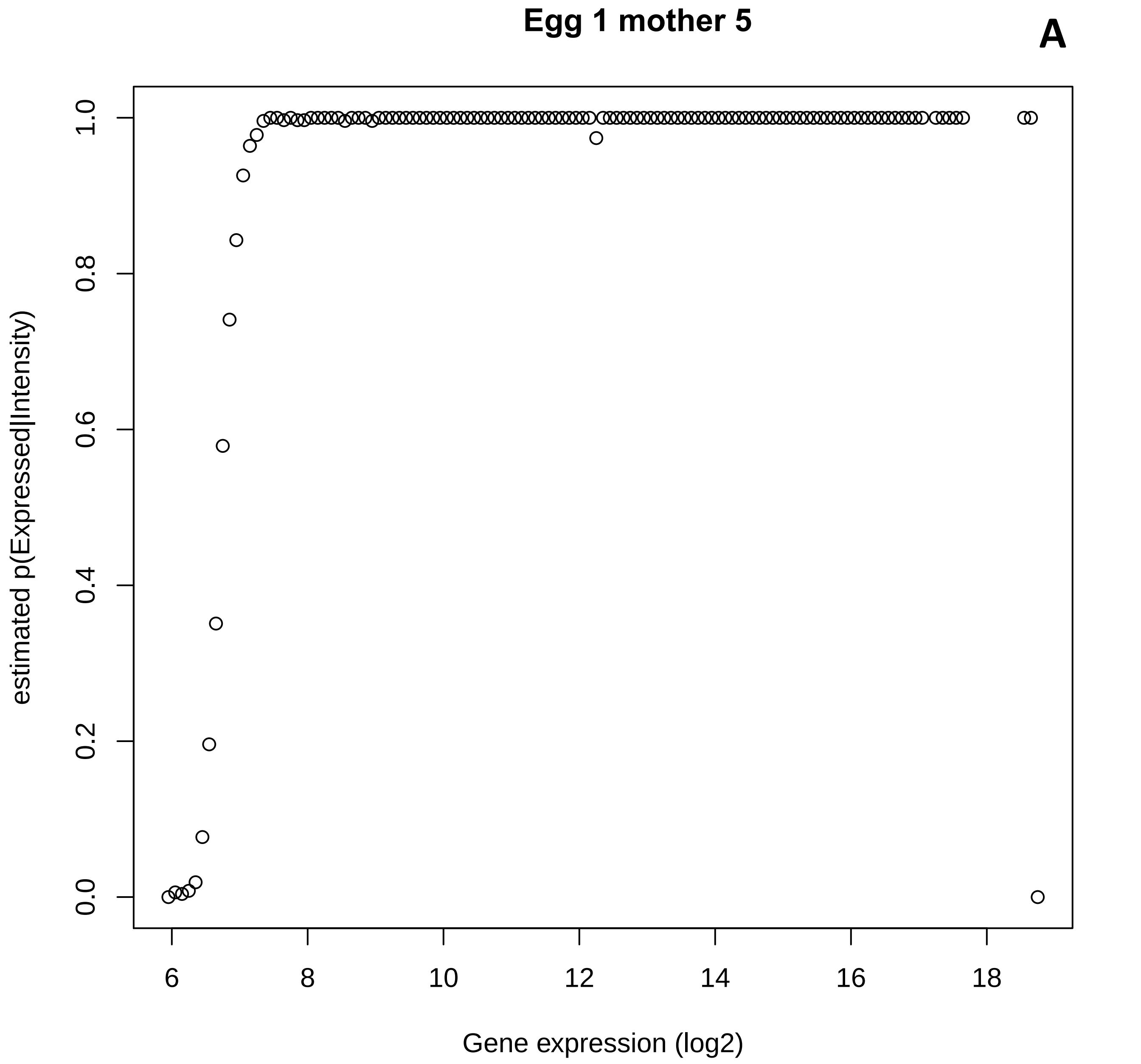

Egg 1 mother 5
A
estimated p(Expressed|Intensity)
0.0
0.2
0.4
0.6
0.8
1.0
6
8
10
12
14
16
18
Gene expression (log2)

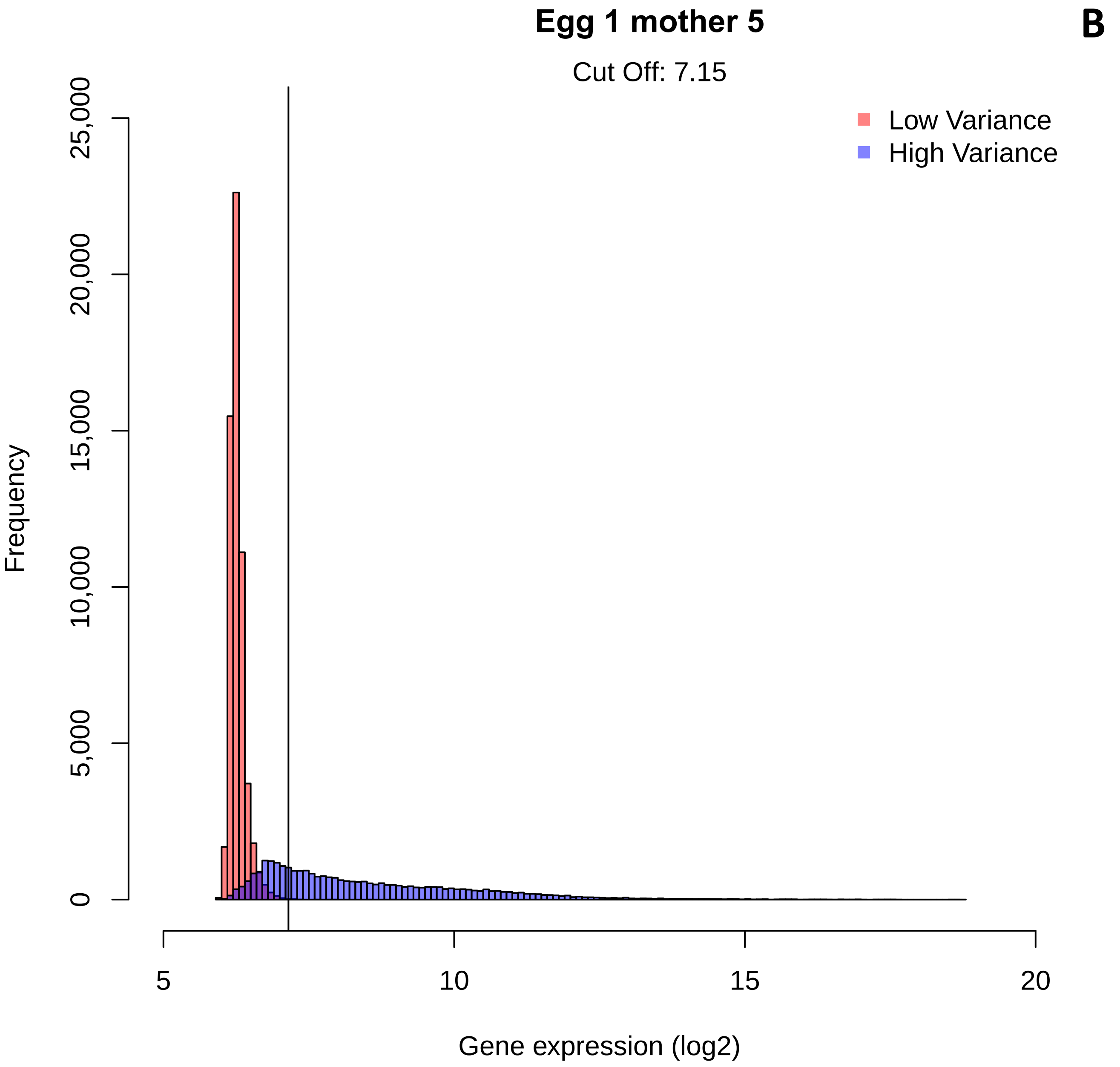

Egg 1 mother 5
B
Cut Off: 7.15
Low Variance
High Variance
Frequency
0
5,000
10,000
15,000
20,000
25,000
5
10
15
20
Gene expression (log2)

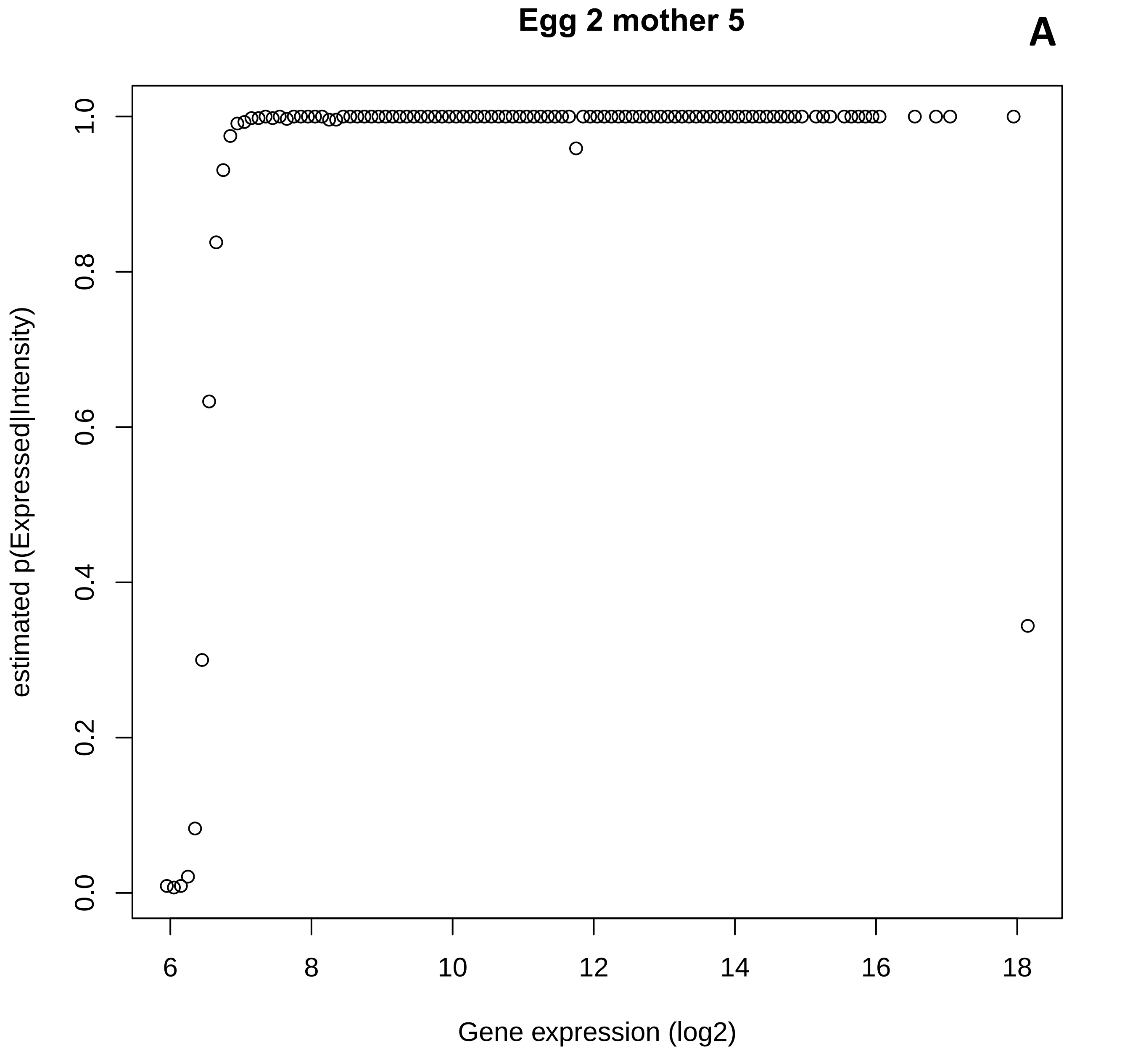

Egg 2 mother 5
A
estimated p(Expressed|Intensity)
0.0
0.2
0.4
0.6
0.8
1.0
6
8
10
12
14
16
18
Gene expression (log2)

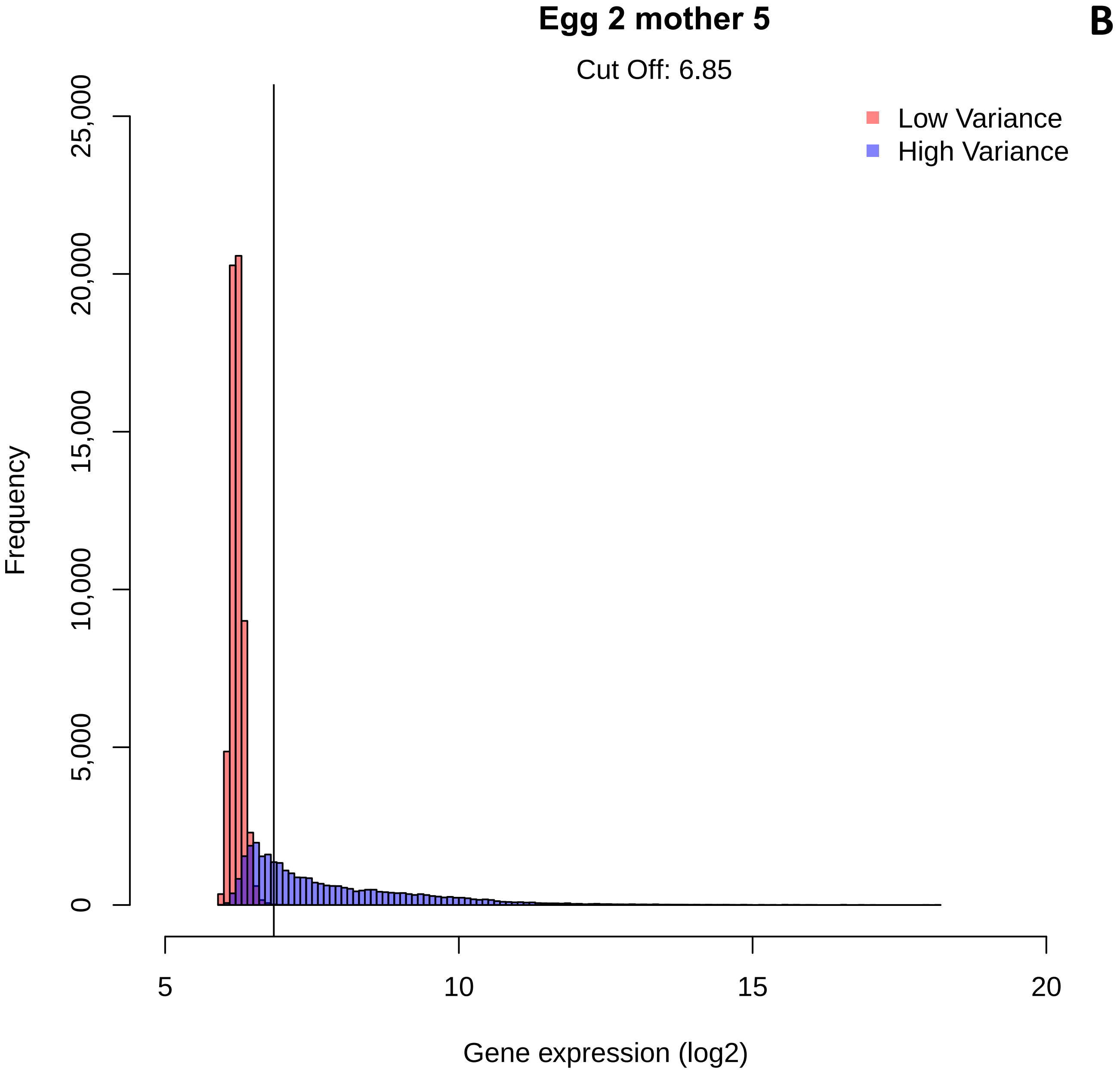
Egg 2 mother 5
B
Cut Off: 6.85
Low Variance
High Variance
Frequency
25,000
20,000
15,000
10,000
5,000
0
5
10
15
20
Gene expression (log2)

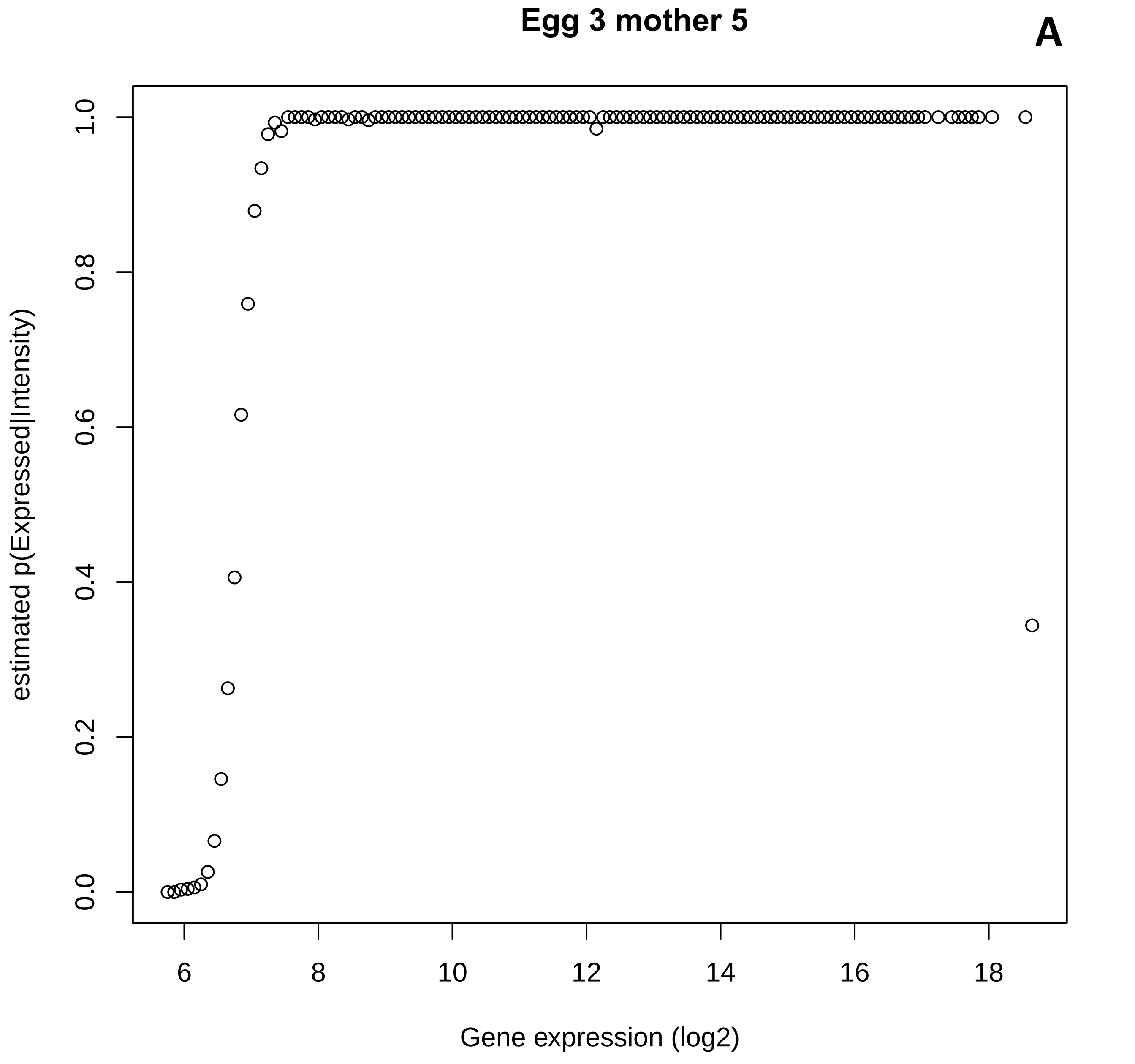

Egg 3 mother 5
A
estimated p(Expressed|Intensity)
0.0
0.2
0.4
0.6
0.8
1.0
6
8
10
12
14
16
18
Gene expression (log2)

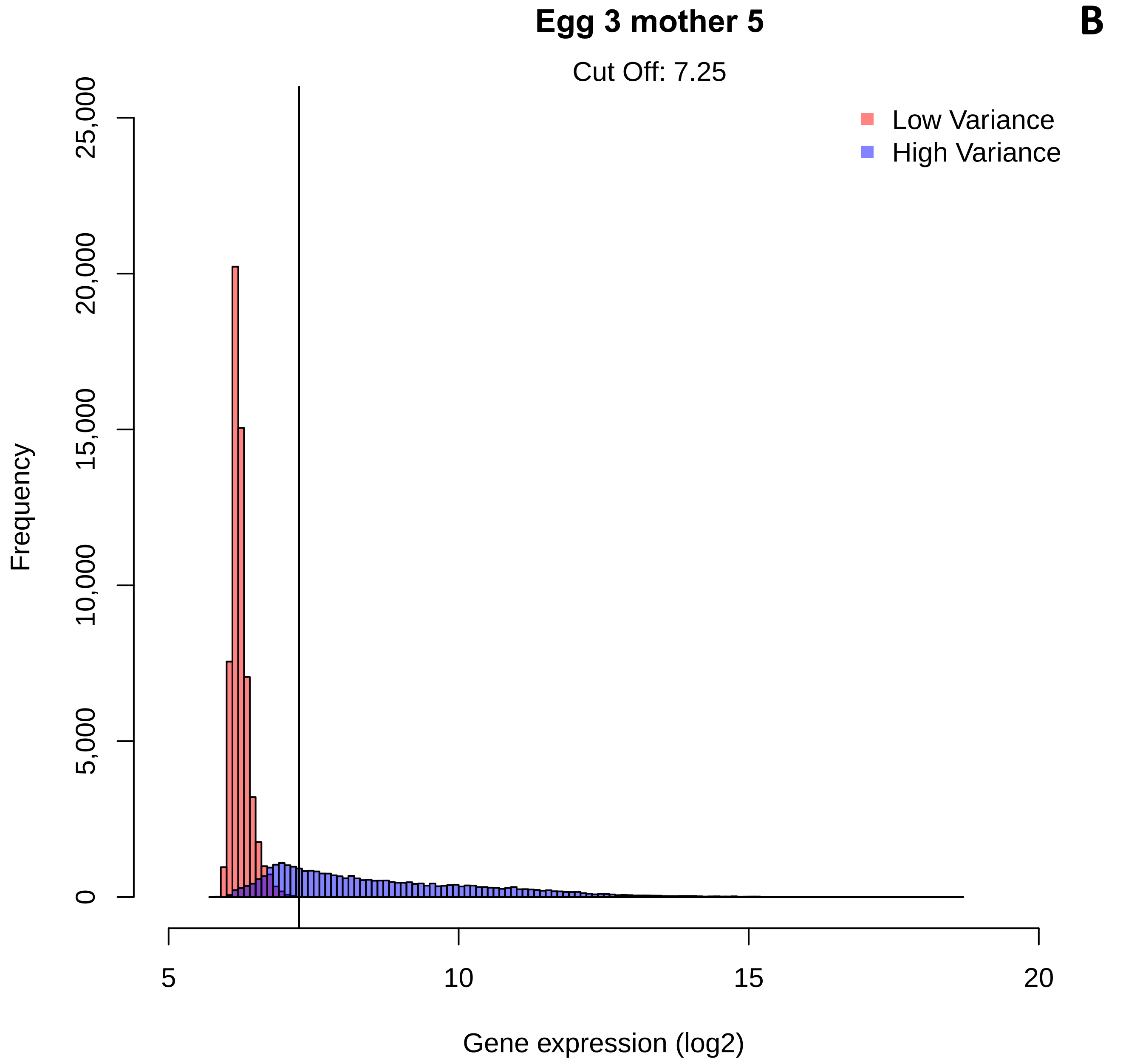

B
Egg 3 mother 5
Cut Off: 7.25
Low Variance
High Variance
Frequency
0
5,000
10,000
15,000
20,000
25,000
5
10
15
20
Gene expression (log2)

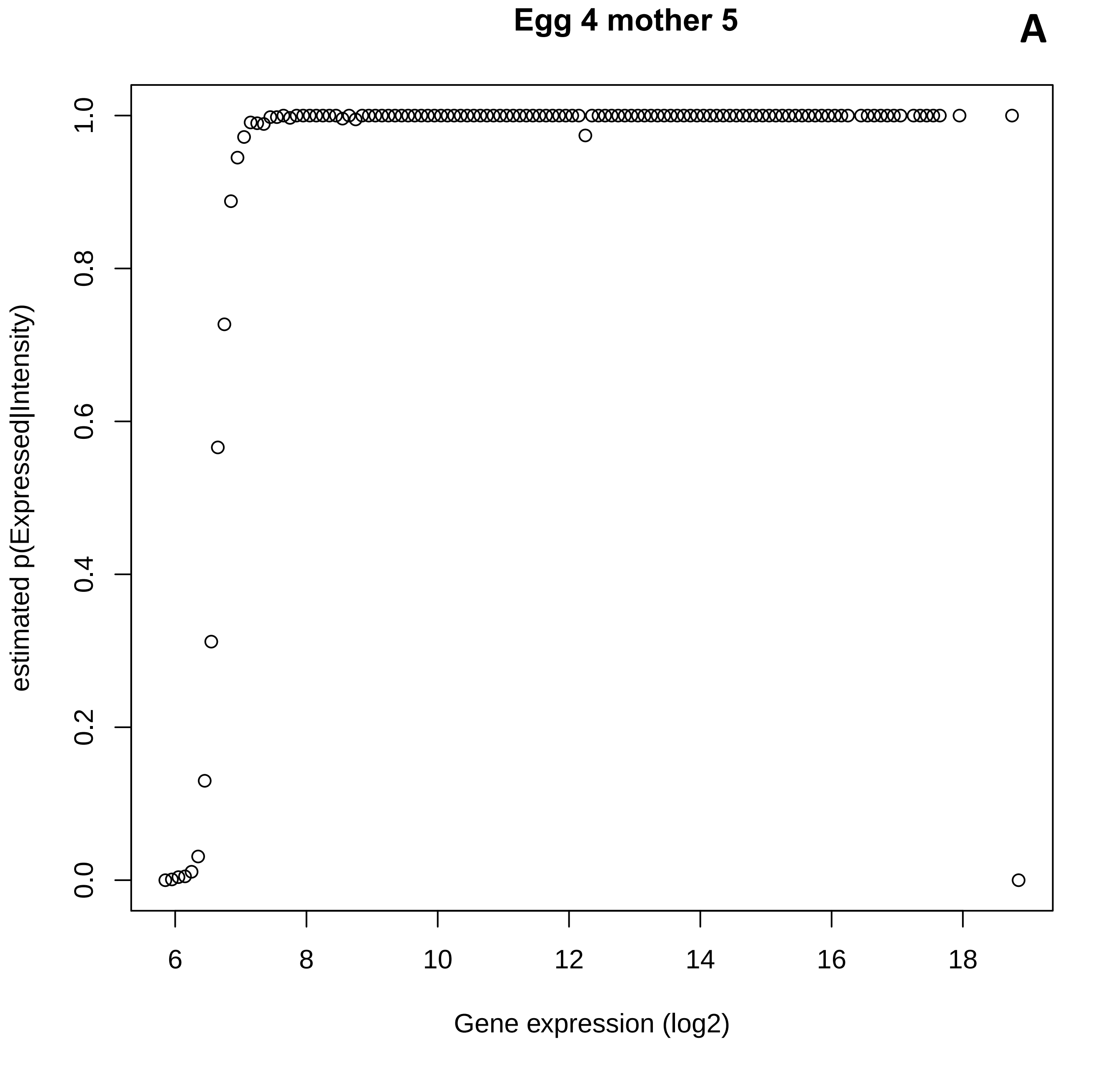

Egg 4 mother 5
A
estimated p(Expressed|Intensity)
0.0
0.2
0.4
0.6
0.8
1.0
6
8
10
12
14
16
18
Gene expression (log2)

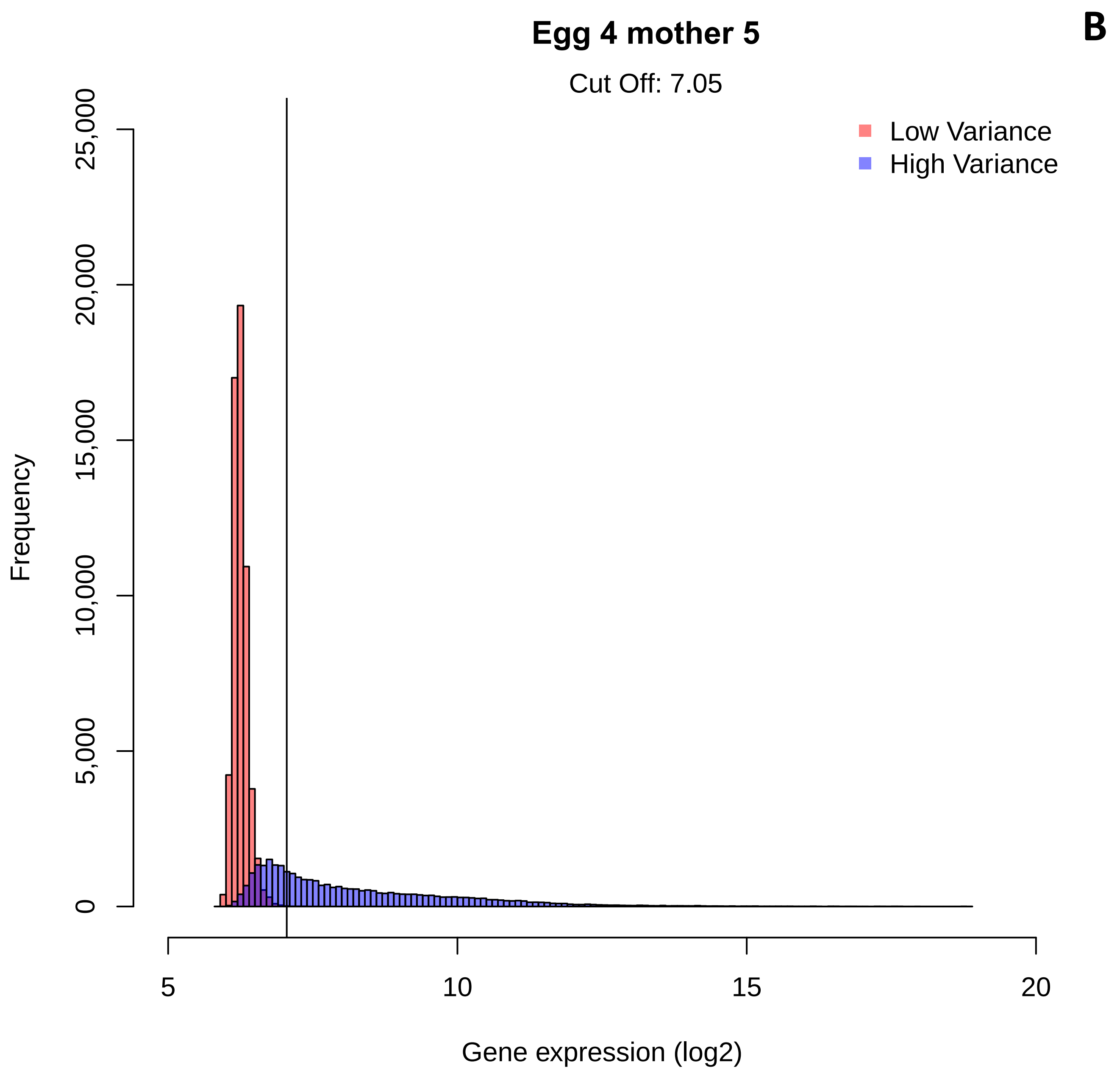
B
Egg 4 mother 5
Cut Off: 7.05
Low Variance
High Variance
Frequency
0
5,000
10,000
15,000
20,000
25,000
5
10
15
20
Gene expression (log2)
