# Supplementary material for: Transcriptome data on maternal RNA of 24 individual zebrafish eggs from five sibling mothers
Source: Data Brief. 2016 Apr 26;8:69–72. doi: 10.1016/j.dib.2016.04.045 (PMC4887590; doi:10.1016/j.dib.2016.04.045)
Supplement: Supplementary file 4 — Supplementary material [file mmc4.pdf]

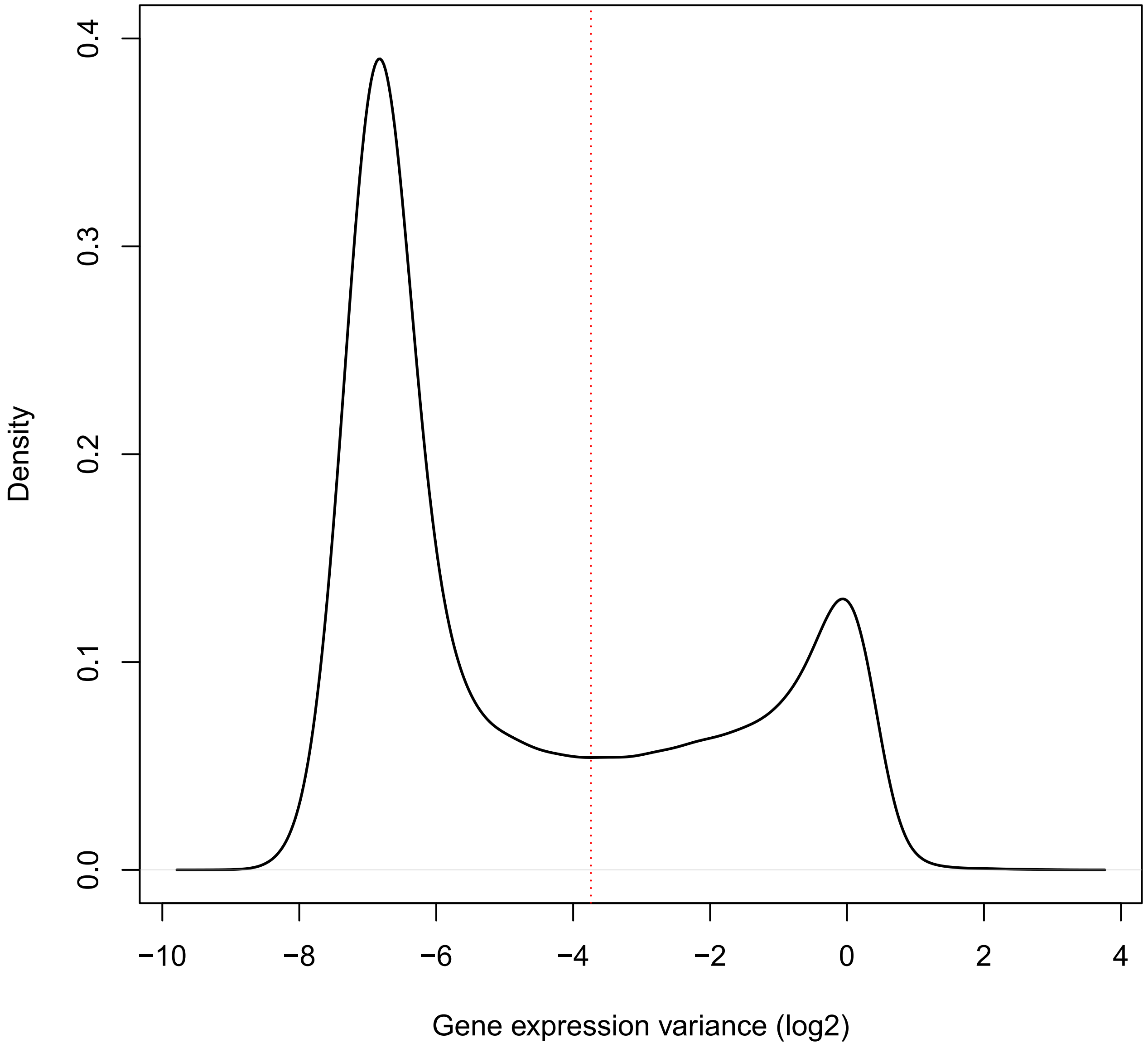

Supplemental Figure SF1
Distribution of the gene-expression intensity variance of raw expression data. The variance cut-off is indicated by a dotted red vertical line at a log2 variance at -3.74.

.
